# The association between type of conception through medically assisted reproduction and childhood cognition: a Danish population-wide cohort study

**DOI:** 10.1093/eurpub/ckad123

**Published:** 2023-07-22

**Authors:** Peter Fallesen

**Affiliations:** Swedish Institute of Social Research, Stockholm University, Stockholm, Sweden; ROCKWOOL Foundation, København K, Denmark

## Abstract

**Background:**

Previous research has indicated that children conceived through medically assisted reproduction (MAR) generally have cognitive outcomes comparable to or better than naturally conceived children. However, previous studies have been limited in their ability to examine this relationship at a population level and consider variations across different types of MAR.

**Methods:**

This study utilizes data from all live births in Denmark between 2006 and 2009 (*n* = 259 608), including a subset of births resulting from MAR conceptions (*n* = 13 566). The dependent variable is the standardized test scores obtained in the second and third grades of primary schools. A comparison is made between the test scores of children spontaneously conceived (SC) and those conceived through intrauterine insemination (IUI) and assisted reproductive technologies (ART). Ordinary least squares regressions are employed, with a baseline model adjusted only for birth year, as well as models that additionally account for conception-related confounders and sociodemographic family characteristics.

**Results:**

In the baseline analysis, ART- and IUI-conceived children displayed better test scores compared with their SC peers. However, after adjusting for relevant factors, ART-conceived children performed worse than SC peers, while IUI-conceived children performed equally well as SC peers and better than ART-conceived children.

**Conclusions:**

These results likely reflect differences in the selection process of potential parents into the type of MAR, as well as the consequences of variations in fecundability. Nevertheless, the differences observed across conception types were overshadowed by test score disparities in socioeconomic background.

## Introduction

With the ongoing rise in maternal age at birth across the global north^1^ and the suggested decline in human fecundity,^2^ births occurring following medically assisted reproduction (MAR) have consistently increased in the 21st century.^3^ In 2018, 9.8% of children born in the frontrunner country Denmark were conceived following some type of MAR treatment.^4^ With birth rates continuing to decline, MAR is poised to play an increasingly important role in upholding fertility levels.^5^ Furthermore, with rises in singlehood^6^ and same-sex families^7^ also occurring in recent decades, women whose sole barrier to motherhood is the absence of a male partner are likely to increasingly seek MAR treatment.

The growth in MAR births has motivated an expanding literature that considers the consequences of MAR conceptions for children’s well-being and development. Children conceived through MAR have poorer birth outcomes,^8–10^ but studies of later-life well-being and cognitive ability in child- and adulthood find null or positive association.^11–19^ However, while studies using population-wide data materials have been conducted for the association between MAR conception and birth outcomes,^8^^,^^9^ the literature studying the associations with cognitive ability and well-being has been conducted on more selective cohort samples, which raises questions about external validity.^18^ Most population-wide studies conducted are somewhat dated and focus on rarer events, such as mental disorders,^20^^,^^21^ or study only a subset of MAR treatment types in low-prevalence countries.^17^^,^^22^ No previous study has examined the population-wide association between MAR conception and cognitive ability, nor considered the role of the type of MAR treatment.^18^

Denmark provides the optimal case study for considering the population-wide consequences of MAR births. Since 2006, MAR treatment has been made available in Denmark to all women, regardless of sexual orientation and partnership status. Furthermore, public funding is available for three cycles of treatment if childlessness is due to infertility, it is the woman's first child with the present partner, the body mass index (BMI) is below 35 (30 if older than 35), the woman is younger than 40, and the woman is considered mentally and physically capable to care for a child. Therefore, non-biological barriers to MAR conception are lower in Denmark than in most if not all other countries, which is also reflected in MAR births accounting for close to 10% of all births in Denmark.^4^

The present study has two aims. First, it examines the association between MAR conception and mid-childhood cognitive development using population-wide data from four Danish birth cohorts born between 2006 and 2009 (*n* = 259 608). Second, the study examines if there is heterogeneity in cognitive ability across types of MAR conception.^18^ The study distinguishes between conceptions following assisted reproductive technologies (ART), which includes all treatments in which either eggs or embryos are handled, and conceptions following intrauterine insemination (IUI).

## Methods

### Study population

**Figure 1:**
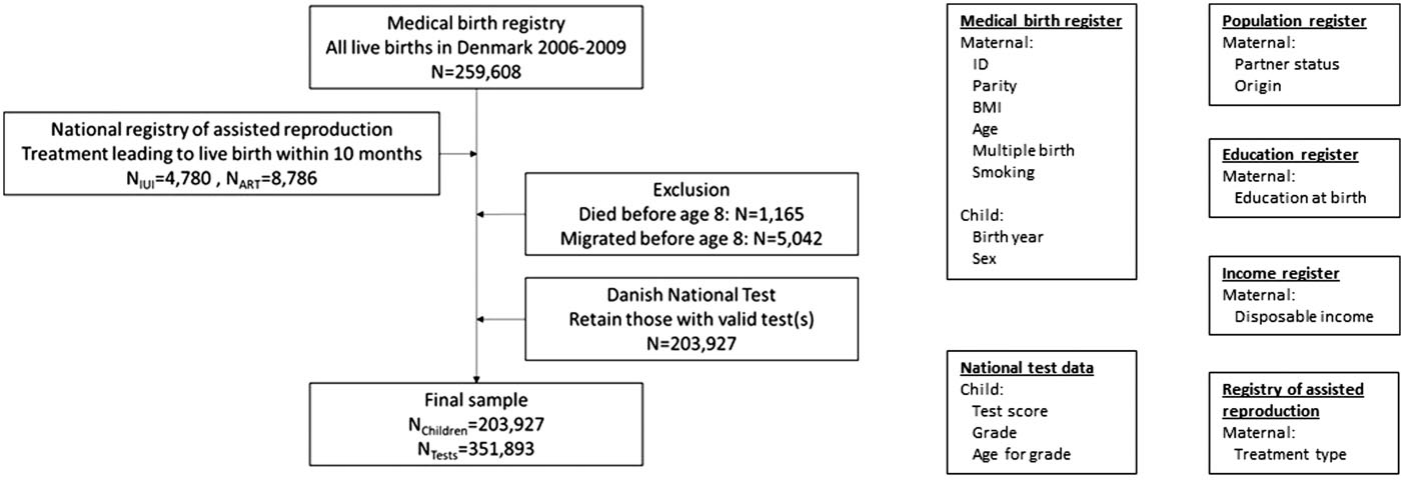
Overview of data structure and variables

I used four birth cohorts consisting of all live births in Denmark between 1 January 2006 and 31 December 2009 drawn from the Danish Medical Birth Register,^23^ which includes all births in hospitals and from in-home deliveries. The data include information on the unique personal identifiers of the mother and child, as well as information on maternal and offspring health. Through the unique personal identifier, I linked mothers to the Danish National Register of Assisted Reproductive Technology,^24^ which includes information on the type and timing of MAR treatments carried out in Denmark at public and private clinics. The data contain all ART treatments and a subset of IUI treatments, as not all private clinics reported IUI treatments prior to 2010. To obtain information on sociodemographic and socioeconomic characteristics, I further linked mothers to the Danish Population,^25^ Education^26^ and Income^27^ registers. Finally, I linked children to their second- and third-grade test scores using the register for the Danish National Tests.^28^Figure 1 gives an overview of the data structure.

The gross study population includes 259 608 live births, of which 13 566 (5.2%) occurred within 10 months of the mother receiving any form of MAR treatment. I exclude children who died (*n* = 1165) or migrated (*n* = 5042) before the age of 9, which is the age children normally take the grade 2 test. Of the remaining study population (*n* = 203 927), 80.8% of children had at least one valid test score. MAR-conceived children were 2.0 percentage points less likely to have a valid test score than spontaneously conceived (SC) children (see Supplementary table SA1). However, after controlling for observable characteristics, the difference in the probability of being in the study population declined to 0.8 percentage points less. Thus, ART-conceived children are less likely to be in the estimation population, but the differences nearly disappeared once adjusted for covariates.

### Variables

#### Outcome variable

As a measure of cognitive development, I use results from low-stakes standardized tests administered in all Danish public schools and some private schools in the second grade (test scores available for all four cohorts, covering 77.5% of cohort members) and the third grade (test scores available for cohorts born between 2006 and 2008, covering 76.9% of cohort members). The national tests were introduced in 2009 for all public schools (with an option to opt-in for private schools, although distinguishing between private and public schools is not possible in the data) and aimed to provide a uniform measure of child school performance in Denmark. The tests are conducted in the spring season and consist of a battery of different tests varying across grades. I focus on the Danish reading comprehension test conducted in grade 2 and the Math ability test conducted in grade 3. The tests are mandatory for all children enrolled in Danish public schools, adaptive and conducted on computers with automatically generated results.^29^

As mentioned, the data do not fully cover children attending private schools. On average, private school children perform as well as, or better than, public school children and come from more advantaged social backgrounds.^30^^,^^31^ Given that MAR births are also more common among socioeconomically advantaged mothers,^32^ this means that students who did not participate in the test can likely expect to have above-average performance. The test results are not displayed in any formal school diplomas and have no direct implications for the child's further school opportunities (there is no tracking or ability grouping in Danish primary or lower secondary schools). The test scores are standardized to have a mean of 0 and an SD of 1 within each grade and year for all children taking the test (not just for the analytical study population), following the approach described by Beuchert and Nandrup.^28^^,^^29^

#### Main independent variable of interest

My indicator for conception following MAR treatment includes all live births that occurred within 10 months of an administered IUI or ART treatment cycle. ART includes intracytoplasmic sperm injection, *in vitro* fertilization (IVF), vitrified-warmed blastocyst replacement/frozen embryo replacement, and oocyte donation. IUI includes inseminations occurring only inside the uterus. In the case of multiple treatments occurring within 10 months of live birth, I use the last recorded treatment.

#### Confounders

I adjust the analyses for a series of confounders known to correlate with the probability of MAR treatment and children's test scores. I divide these confounders into two groups: (i) conception- and pregnancy-related characteristics and (ii) socioeconomic and demographic characteristics.

Conception and pregnancy-related characteristics include a birth year indicator, multiple birth indicator (binary), parity (categorical, with multiple births coded with similar parity), maternal BMI (categorical), maternal smoking during pregnancy (binary) and child sex (binary). These variables were obtained from the medical birth records. In case of missing information, a separate category was created to capture this. I discuss missing values in more detail in the Results section.

Socioeconomic and demographic characteristics include maternal age (categorical), whether the mother was married/cohabiting with a man/woman at the start of the year of birth (binary), maternal level of education at the start of the year of birth (categorical), disposable income in €1000 at the start of the year of birth (continuous), maternal migration background (categorical) and whether the child was young/old for grade when sitting the test (categorical) with ‘old for grade’ including both grade retention and late starters. Grouping details and distribution of confounders are presented in detail in the first part of the Results section.

### Statistical models

I estimate three linear regression models for each grade, with standard errors clustered at the maternal level. First, a baseline model where I only adjust test scores for the birth year. Second, a model where I adjust for the birth year and conception and pregnancy-related confounders. Third, a model where I adjust for the birth year, conception and pregnancy-related, and socioeconomic and demographic confounders. I present separate estimates for ART and IUI, as well as estimates for the difference between ART and IUI. All analyses were conducted using Stata Statistical Software: Release 17 (StataCorp LP, College Station, TX, USA).

## Results

### Descriptive results

Table 1 presents the descriptive statistics of the study population for all births and by mode of conception (SC, ART and IUI). There are fewer children in the study population born in 2009 than in previous years because I only have grade 3 test scores for birth cohorts 2006–08. For both types of MAR, children are more likely to be born in the later included birth years compared with SC children. ART-conceived children are less likely to be male compared with SC children, whereas IUI-conceived children are more likely to be male. Furthermore, ART-conceived children were a factor of 10 (30.7%) more likely to be part of multiple births than SC children (3.1%), and IUI-conceived children (21.3%) a factor of 7.

**Table 1:** Descriptive statistics across conception mode

	ART	IUI	SC	Total
Grade = 2	59.0%	60.2%	57.1%	57.2%
Child male = 1	48.4%	52.1%	50.6%	50.6%
Birth year				
2006	13.6%	9.4%	30.7%	29.8%
2007	33.9%	32.1%	28.8%	29.0%
2008	36.5%	39.5%	27.6%	28.0%
2009	16.0%	19.0%	12.9%	13.1%
Multiple births	30.7%	21.3%	3.1%	4.3%
Maternal age (years)				
<18	NA	NA	0.3%	0.3%
18–24	1.6%	2.4%	12.0%	11.5%
25–29	19.4%	23.2%	32.7%	32.1%
30–34	45.3%	44.3%	37.4%	37.8%
35–39	27.3%	25.0%	15.0%	15.6%
40+ years	6.5%	5.2%	2.7%	2.8%
Parity				
1	62.8%	62.4%	42.5%	43.5%
2	28.8%	30.0%	36.1%	35.7%
3	4.1%	4.8%	13.9%	13.4%
4+	1.6%	0.7%	4.7%	2.9%
Unknown	2.8%	2.1%	2.9%	2.9%
Maternal BMI				
<18.5	6.5%	7.3%	7.9%	7.9%
18.5–24.9	60.4%	58.3%	57.6%	57.7%
25.0–29.9	21.2%	19.9%	19.5%	19.6%
30.0–34.9	7.3%	8.8%	7.3%	7.4%
35.0–39.9	1.5%	3.0%	2.6%	7.4%
40+	0.5%	0.9%	1.3%	1.3%
Unknown	2.6%	1.9%	3.7%	3.7%
Maternal smoking	7.2%	7.4%	14.8%	14.4%
Maternal partner = 1	96.7%	91.7%	88.7%	89.0%
Maternal education				
Lower secondary	9.5%	9.7%	22.1%	21.5%
Upper secondary	38.7%	37.0%	40.3%	40.2%
Tertiary	51.8%	53.3%	37.6%	38.3%
Maternal origin				
Danish native	92.4%	93.4%	87.5%	87.8%
1st gen immigrant	6.9%	5.6%	10.8%	10.6%
2nd gen immigrant	0.5%	1.0%	1.3%	1.2%
Unknown	0.1%	0.1%	0.4%	0.4%
Disposable income	24.872 (10.367)	24.947 (9.490)	21.483 (31.117)	21.650 (30.439)
Child’s age for grade				
Young for grade	1.1%	1.6%	1.7%	1.7%
Aged for grade	91.3%	90.9%	91.7%	91.6%
Old for grade	7.6%	7.5%	6.6%	6.7%
Observations	11 169	5991	334 733	351 893

*Note*: Lower secondary education: ISCED <3. Upper secondary education: ISCED = {3,4}. Tertiary education: ISCED >4. Income measured in €1000. Standard deviation in parentheses.

ART, assisted reproductive technology; BMI, body mass index; ISCED, International Standard Classification of Education; IUI, intra-uterine insemination; SC, spontaneously conceived.

In terms of conception-related maternal characteristics, ART mothers were older (33.8% aged 35 or older) than IUI mothers (30.2% aged 35 or older), who, in turn, were older than mothers of SC children (17.7% aged 35 or older). Both types of MAR-conceived children were also more likely to be their mother’s first birth event (ART: 62.8%, IUI: 62.4%, SC: 42.5%). The shares with missing parity information due to mothers not receiving full prenatal care in Denmark (expats, migrants and tourists) and misregistration were similar across conception type (ART: 2.8%, IUI: 2.1%, SC: 2.9%). Mothers of ART children had a lower BMI (88.1% had BMI < 30) than mothers of SC children (85.0% had BMI < 30), whereas differences between SC and IUI (IUI: 85.5% had BMI < 30) were more muted. A percentage of 3.7 of the study population reported missing maternal BMI. Of these, 2.9% were missing due to no registration of maternal BMI because the mother was not present in Denmark during the start of the pregnancy (e.g. expats coming home to give birth, and expats and migrants pregnant upon arrival in Denmark). For the remaining 0.9%, this was due to reporting an implausible value of either height or weight. MAR mothers were half as likely to smoke during pregnancy (ART: 7.2%, IUI: 7.4%, SC: 14.8%).

In terms of socioeconomic and demographic characteristics, MAR mothers had higher levels of education (51.8% of ART had a tertiary degree, 53.3% of IUI, and 37.6% for SC), were less likely to have a migration background (92.4% of ART were Danish natives, 93.4% of IUI and 87.5% for SC, with a small amount having missing information) and had higher disposable income (post-tax and transfers). Lastly, MAR-conceived children were slightly more likely to have started school late or have been held back, and thus be old for the grade (ART: 7.6%, IUI: 7.5%, SC: 6.6%).

### Regression results

**Figure 2:**
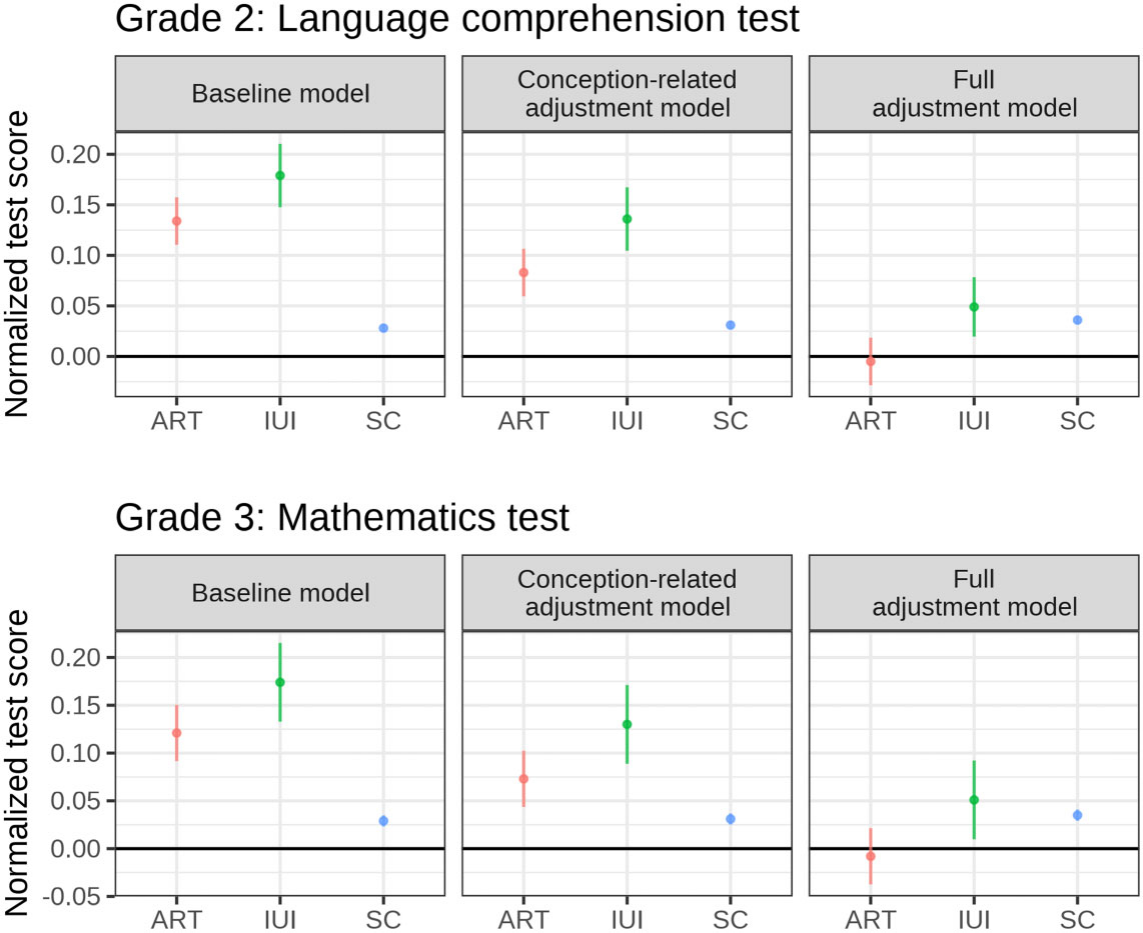
Predictive margins for normalized test scores and 95% confidence intervals by mode of conception. Standard errors clustered at maternal level. Baseline: adjusted for birth year. Conception-related adjustment: adjusted for birth year, multiple birth, parity, maternal BMI, maternal smoking and child sex. Full adjustment: adjusted for birth year, multiple birth, maternal age, parity, maternal BMI, maternal smoking, maternal relationship status, maternal education, maternal origin, maternal disposable income, child young (old) for grade and child sex. ART, Assisted reproductive technology; BMI, body mass index; IUI, intrauterine insemination; SC, spontaneously conceived.

Figure 2 presents the predictive margins and 95% confidence intervals (CI) at the mean value of adjustment variables across conception modes, models and grades of the test from regressing the within-grade-and-year population standardized test score on mode of conception per grade. The full model results are presented in Supplementary tables SA2 and SA3. It should be noted that the test scores are grade- and year-standardized within the full population of all children taking the test, and not just children born in Denmark within the birth years considered, which is why the predictive margins in figure 2 tend to all be larger than 0. Estimates are directly interpretable as a share of a standard deviation.

Left panel report the estimates from the baseline model only adjusted for birth year. ART- and IUI-conceived children have higher standardized test scores than SC children in grade 2, with ART-conceived children having 0.106 SD (95% CI: 0.081–0.130) higher test scores, whereas IUI-conceived children had 0.151 SD (95% CI: 0.119–0.182) higher test scores. The same pattern was found in grade 3, with ART having 0.092 SD (95% CI: 0.063–0.121) higher and IUI having 0.121 SD (95% CI: 0.103–0.187) higher scores. Furthermore, differences between IUI and ART were also statistically detectable with 95% CIs above zero for both grades.

After adjusting for birth- and conception-related characteristics in middle panels, the higher standardized test scores were somewhat attenuated, but both ART- and IUI-conceived children still had higher standardized test scores than SC children, and the differences between ART and IUI increased.

Lastly, after adjusting also for socioeconomic and demographic characteristics in right panels, ART-conceived children had lower test scores than SC children in grade 2 (−0.040 SD, 95% CI: −0.064 to −0.016) and grade 3 (−0.043 SD, 95% CI: −0.072 to −0.014). For IUI-conceived children, there was no detectable difference against SC children in grade 2 (0.014 SD, 95% CI: −0.016 to 0.045) and grade 3 (0.016 SD, 95% CI: −0.025 to 0.057). The difference between ART- and IUI-conceived children remained similar in size to those found in previous models (grade 2: 0.055 SD, 95% CI: 0.017–0.092; grade 3: 0.059 SD, 95% CI: 0.010–0.108).

**Figure 3:**
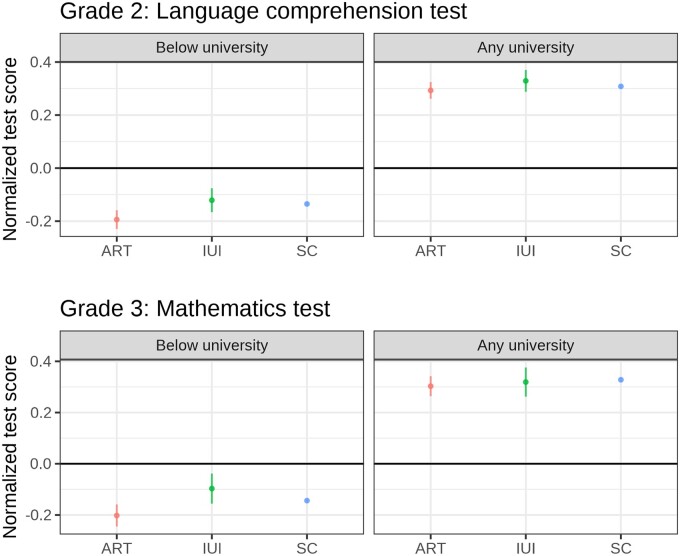
Predictive margins for normalized test scores and 95% confidence intervals by mode of conception and maternal educational level at birth. Standard errors clustered at maternal level. Full adjustment: adjusted for birth year, multiple birth, maternal age, parity, maternal BMI, maternal smoking, maternal relationship status, maternal origin, maternal disposable income, child young (old) for grade and child sex. ART, Assisted reproductive technology; BMI, body mass index; IUI, intra-uterine insemination; SC, spontaneously conceived.

As a first sensitivity analysis, I divided the study population by whether the mother had a university degree or not to take the educational gradient in MAR usage into account. Figure 3 presents the predicted margins from the fully adjusted model (full set of parameters is shown in Supplementary table SA4). Differences in test scores across conception modes are larger among mothers with education below university than among mothers with any university education. However, differences in test scores between modes of conception are vastly overshadowed by differences in test scores by maternal education.

For the rest of the sensitivity tests, an overview figure is reported in Supplementary figure SA1 as well as tables of estimates. As the second test, the study population was limited to only include singleton births because multiple births are more common following MAR conception and found to have lower academic achievement.^33^Supplementary tables A5 and A6 report the regression coefficients from this sensitivity analysis. The patterns across conception types remained the same, although the difference between ART- and IUI-conceived children attenuated. As a third sensitivity analysis, the study population was limited to only include first births. This was done because first births are overrepresented among MAR-conceived children (see table 1) and birth order is associated with a test score advantage.^34^Supplementary tables SA7 and SA8 report the results from this sensitivity analysis. The patterns across conception types remained the same, and the difference between ART- and IUI-conceived children was even larger. As the fourth test, only children born after 2006 were considered (because single and lesbian women only got legal access to treatment in 2006). Supplementary tables SA9 and SA10 report these findings. Differences between ART and IUI became a bit attenuated, but the pattern remained, and remained statistically detectable.

## Discussion

In this study, I used population-wide data for four Danish birth cohorts to examine the association between conception type (ART, IUI or SC) and children's test scores in grades 2 and 3. My results showed that although ART- and IUI-conceived children had higher test scores than SC children in unadjusted models, these differences mostly disappeared after adjusting for birth, socioeconomic and sociodemographic characteristics. In fact, ART-conceived children were found to perform below both SC and IUI-conceived children.

The study expands the existing literature on cognitive development in MAR-conceived children by utilizing population-wide data from a context with high usage and easy availability of MAR treatment^35^ and smaller variation in early-life care and school quality compared with most other countries.^36^^,^^37^ Previous research has mainly relied on data from settings with lower MAR uptake and higher educational inequality, such as the UK,^11^^,^^15^ Sweden^17^ and Australia.^22^ By focusing on Denmark, where MAR-conceived births now constitute close to 10% of all births, I have provided a vanguard country perspective on the relationship between MAR conception and children's academic achievement.

The second key finding is that ART-conceived children consistently had lower test scores than IUI-conceived children, even after controlling for various factors. One possible explanation, although not explored in this study, is that parents conceiving through ART may have lower fecundability compared with those conceiving through IUI. This could also account for the lower sex ratio in ART-conceived children, as it is known to decline under harsher conditions.^38^ Furthermore, the fact that ART mothers are more likely to be in a relationship and older than IUI mothers suggests a longer period until successful conception, which could indicate low fecundability. Recent work by Magnus *et al*.^39^ has linked parental sub-fecundability to neurodevelopmental difficulties and delays in children, which could explain the gradient in children's test scores within the MAR treatment group.

There are several limitations of the present study that should be noted. First, I only observe MAR conceptions that occurred in Denmark, and I do not have data on the full universe of Danish IUI treatments. Therefore, women who exhausted their cycles or were ineligible for public funding for MAR and subsequently conceived at clinics outside Denmark would be misclassified as SC, along with unreported Danish IUI treatments. However, any bias resulting from this misclassification would likely drive the estimates towards 0. Second, a key variable of interest that I do not observe is the time from the first conception attempt to the successful conception. Having this information would have allowed for a more comprehensive consideration of the role of fecundability. Third, MAR-conceived children were slightly less likely to participate in the test, but as discussed earlier, this likely introduced a bias towards 0. Fourth, the definition of MAR conception (any treatment within 10 months of birth) leaves room for some children to be SC, which could bias the estimates towards the reference category. Fifth, I do not have data on children's cognitive ability earlier in childhood, leaving more time for environmental influences to compensate for any initial developmental difficulties. Last, the analyses did not consider mediating roles of prematurity/low birth weight and developmental disorders, which are more prevalent among MAR-conceived children.^9^^,^^12^^,^^13^^,^^20^ This final limitation was by design—insofar as children remain in the sample even when having poorer birth or developmental outcomes, adjusting for such mediators could induce additional issues of confounding that would make the associations between MAR and test score more difficult to interpret. Further, although one could worry about selection out of sample due to these children having a higher risk of attending special institutions exempt from participating in the national test, only 4% of Danish second and third graders attending special educational schools. These children are most likely to come from low education and low-income families,^40^ which in turn are the families least likely to conceive through MAR (see table 1).

Nevertheless, the study also has significant strengths. Firstly, it utilizes a nationwide multi-cohort dataset, which is likely less biased than other study populations or samples used in previous literature. Additionally, it allows for differentiation between types of MAR treatment. Secondly, I examine a general outcome that applies to all children (test scores) in a standardized and objective manner, rather than focusing on rare neurodevelopmental outcomes, such as psychiatric diagnoses, which could be subject to clinicians' discretion and influenced by knowledge about the child's method of conception.

In conclusion, my findings demonstrate that after adjusting for relevant conception-related and socioeconomic variables, ART-conceived children perform worse than their SC peers, while IUI-conceived children perform as well as SC peers and even better than ART-conceived peers. Therefore, this study contributes new knowledge by revealing that in the country with the highest MAR conception rate globally, there are differences in children's educational achievement depending on the mode of MAR conception. However, these differences, although not negligible, are overshadowed by socioeconomic disparities in educational achievement. Increased usage of ART may lead to a slight decrease in test scores among children, but the magnitude of these differences is minor compared with variations along socioeconomic gradients. Therefore, it is unlikely that increased MAR usage to enhance fertility rates would result in a drastic decline in childhood cognition.

## Supplementary Material

ckad123_Supplementary_Data

## Data Availability

The present study received approval from Statistics Denmark under the auspices of data project no. 705830. Access and use medical records or other material used in the study obtained from Danish Health Data Authority under the same data project. The data underlying this article cannot be shared publicly due to privacy concerns restricting availability of register data for research. The author can make aggregated data available, conditional on ethical vetting. The author accessed the individual-level data through Statistics Denmark online access system. If a researcher at a university or other research institution outside Denmark wishes to use these data, this may be accomplished by visiting a Danish research institution or by cooperating with researchers or research assistants working in Denmark. All scripts to generate data and results underlying the study are available as supplementary materials.
