# Supplementary material for: The association between type of conception through medically assisted reproduction and childhood cognition: a Danish population-wide cohort study
Source: Eur J Public Health. 2023 Jul 22;33(6):1020–6. doi: 10.1093/eurpub/ckad123 (PMC10710341; doi:10.1093/eurpub/ckad123)
Supplement: ckad123_Supplementary_Data [file ckad123_supplementary_data.zip › ckad123_Supplementary_Data/ejph-2023-03-om-0148-File005.docx]

# Supplemental Material

| Figure A1: Predictive margins for normalized test scores and 95% confidence intervals by mode of conception and sensitivity check |
| --- |
| 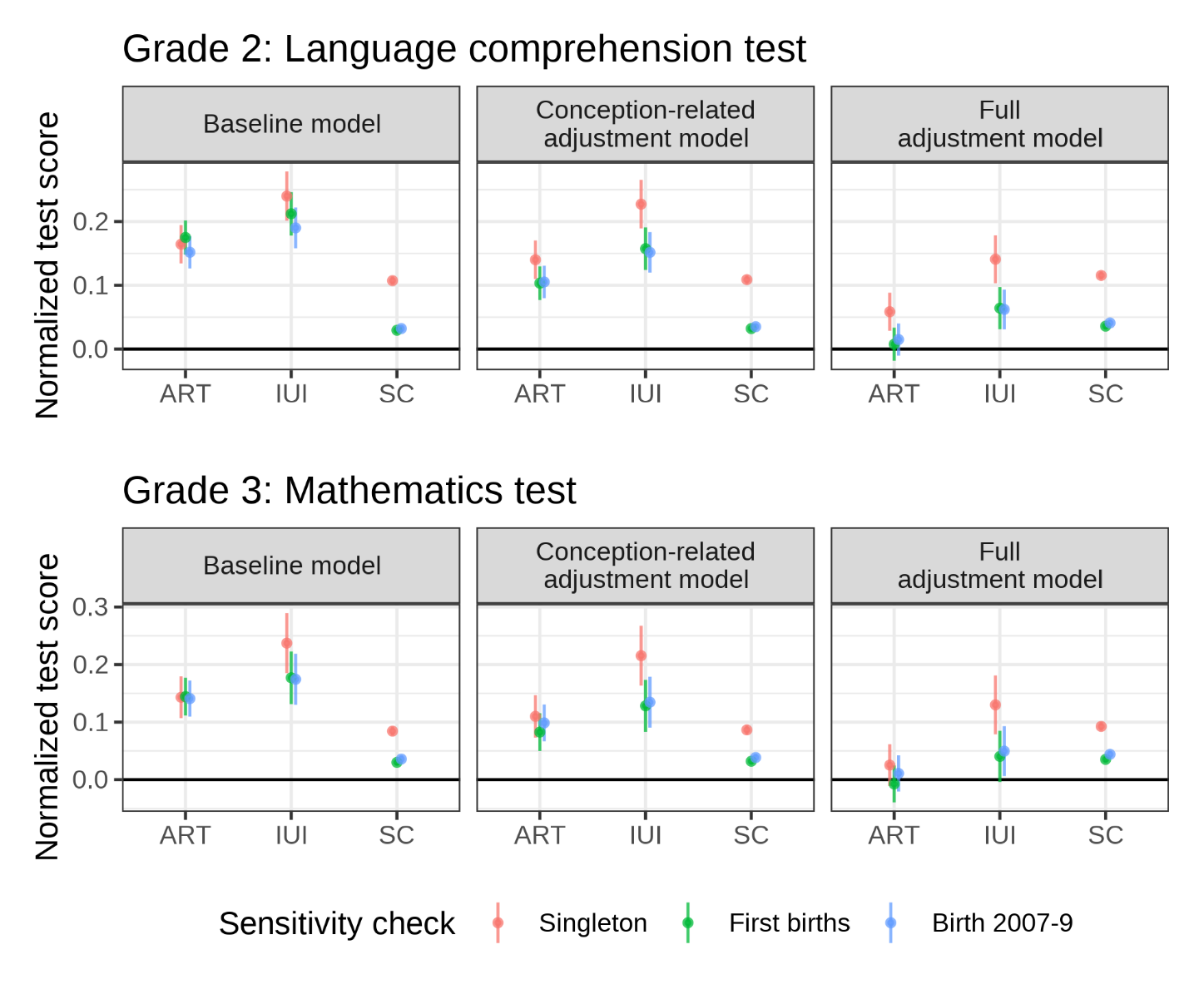 |
| Standard errors clustered at maternal level.  SC: Spontaneously conceived. ART: Assisted reproductive technology. IUI: Intrauterine insemination.  Baseline: Adjusted for birth year. Conception-related adjustment: Adjusted for birth year, multiple birth, parity, maternal body mass index (BMI), and maternal smoking, child sex.  Full adjustment: Adjusted for birth year, multiple birth, maternal age, parity, maternal body mass index (BMI), maternal smoking, maternal relationship status, maternal education, maternal origin, maternal disposable income, child young (old) for grade and child sex. |

| Table A1: Linear model regressing being in the analytical study population on mode of conception and covariates | | | |
| --- | --- | --- | --- |
|  | Baseline | Fully adjusted |  |
| Conceived through MAR=1 | -0.020 | -0.007 |  |
|  | [-0.027,-0.013] | [-0.014,0.000] |  |
| Birth year |  |  |  |
| 2006 | Ref. | Ref. |  |
|  |  |  |  |
| 2007 | -0.014 | -0.012 |  |
|  | [-0.018,-0.009] | [-0.016,-0.008] |  |
| 2008 | -0.026 | -0.023 |  |
|  | [-0.030,-0.021] | [-0.028,-0.019] |  |
| 2009 | -0.110 | -0.106 |  |
|  | [-0.114,-0.105] | [-0.110,-0.102] |  |
| Multiple birth |  | 0.003 |  |
|  |  | [-0.005,0.011] |  |
|  |  |  |  |
| Maternal age |  |  |  |
| <18 years |  | 0.019 |  |
|  |  | [-0.010,0.049] |  |
| 18-24 years |  | 0.029 |  |
|  |  | [0.023,0.035] |  |
| 25-29 years |  | 0.023 |  |
|  |  | [0.019,0.027] |  |
| 30-34 years |  | Ref. |  |
|  |  |  |  |
| 35-39 years |  | -0.037 |  |
|  |  | [-0.041,-0.032] |  |
| 40+ years |  | -0.060 |  |
|  |  | [-0.069,-0.051] |  |
|  |  |  |  |
| Parity |  |  |  |
| 1 |  | Ref. |  |
|  |  |  |  |
| 2 |  | 0.017 |  |
|  |  | [0.013,0.020] |  |
| 3 |  | 0.017 |  |
|  |  | [0.012,0.022] |  |
| 4+ |  | 0.018 |  |
|  |  | [0.010,0.026] |  |
| Unknown |  | -0.041 |  |
|  |  | [-0.051,-0.031] |  |
|  |  |  |  |
| Maternal BMI |  |  |  |
| <18.5 |  | 0.003 |  |
|  |  | [-0.003,0.008] |  |
| 18.5-24.9 |  | Ref. |  |
|  |  |  |  |
| 25.0-29.9 |  | 0.010 |  |
|  |  | [0.006,0.014] |  |
| 30.0-34.9 |  | 0.019 |  |
|  |  | [0.013,0.025] |  |
| 35.0-39.9 |  | 0.008 |  |
|  |  | [-0.002,0.018] |  |
| 40+ |  | 0.027 |  |
|  |  | [0.013,0.041] |  |
| Unknown |  | -0.007 |  |
|  |  | [-0.016,0.002] |  |
|  |  |  |  |
| Maternal smoking |  | 0.028 |  |
|  |  | [0.023,0.032] |  |
|  |  |  |  |
| Maternal partner =1 |  | 0.013 |  |
|  |  | [0.008,0.018] |  |
|  |  |  |  |
| Maternal education |  |  |  |
| Lower secondary |  | Ref. |  |
|  |  |  |  |
| Upper secondary |  | 0.009 |  |
|  |  | [0.005,0.014] |  |
| Tertiary |  | -0.005 |  |
|  |  | [-0.010,0.000] |  |
|  |  |  |  |
| Maternal origin |  |  |  |
| Danish native |  | Ref. |  |
|  |  |  |  |
| 1st gen immigrant |  | -0.048 |  |
|  |  | [-0.053,-0.042] |  |
| 2nd gen immigrant |  | -0.094 |  |
|  |  | [-0.107,-0.081] |  |
| Unknown |  | -0.241 |  |
|  |  | [-0.263,-0.218] |  |
|  |  |  |  |
| Disposable income |  | 0.000 |  |
|  |  | [0.000,0.000] |  |
| Income missing |  | 0.019 |  |
|  |  | [0.000,0.039] |  |
| Child male=1 |  | -0.016 |  |
|  |  | [-0.019,-0.013] |  |
|  |  |  |  |
| Constant | 0.846 | 0.828 |  |
|  | [0.843,0.849] | [0.820,0.835] |  |
| Observations | 252349 | 252349 |  |
| 95% confidence intervals in brackets. MAR: Medically assisted reproduction. BMI: Body mass index. Lower secondary education: ISCED < 3. Upper secondary education: ISCED = {3,4}. Tertiary education: ISCED > 4. . ISCED: International Standard Classification of Education. Income measured in €1,000. | | | |

| Table A2: Linear model regressing second grade language comprehension score on mode of conception and covariates | | | | |
| --- | --- | --- | --- | --- |
|  | Baseline^a^ | Adjusted for birth related^b^ | Fully adjusted^c^ |  |
| ART | 0.106 | 0.052 | -0.040 |  |
|  | [0.082,0.130] | [0.028,0.077] | [-0.064,-0.016] |  |
| IUI | 0.151 | 0.105 | 0.014 |  |
|  | [0.119,0.182] | [0.074,0.137] | [-0.016,0.045] |  |
| Birth year |  |  |  |  |
| 2006 | Ref. | Ref. | Ref. |  |
|  |  |  |  |  |
| 2007 | 0.003 | 0.001 | 0.002 |  |
|  | [-0.009,0.015] | [-0.011,0.013] | [-0.009,0.014] |  |
| 2008 | 0.008 | 0.007 | 0.009 |  |
|  | [-0.004,0.019] | [-0.004,0.019] | [-0.002,0.021] |  |
| 2009 | 0.046 | 0.033 | 0.010 |  |
|  | [0.034,0.058] | [0.022,0.045] | [-0.001,0.022] |  |
|  |  |  |  |  |
| Multiple birth |  | -0.067 | -0.082 |  |
|  |  | [-0.092,-0.042] | [-0.106,-0.058] |  |
|  |  |  |  |  |
| Parity |  |  |  |  |
| 1 |  | Ref. | Ref. |  |
|  |  |  |  |  |
| 2 |  | -0.079 | -0.144 |  |
|  |  | [-0.089,-0.070] | [-0.154,-0.135] |  |
| 3 |  | -0.163 | -0.222 |  |
|  |  | [-0.176,-0.150] | [-0.236,-0.208] |  |
| 4+ |  | -0.407 | -0.362 |  |
|  |  | [-0.431,-0.384] | [-0.386,-0.338] |  |
| Unknown |  | -0.109 | -0.078 |  |
|  |  | [-0.138,-0.079] | [-0.107,-0.050] |  |
|  |  |  |  |  |
| Maternal BMI |  |  |  |  |
| <18.5 |  | -0.145 | -0.054 |  |
|  |  | [-0.162,-0.128] | [-0.070,-0.037] |  |
| 18.5-24.9 |  | Ref. | Ref. |  |
|  |  |  |  |  |
| 25.0-29.9 |  | -0.135 | -0.105 |  |
|  |  | [-0.146,-0.123] | [-0.116,-0.094] |  |
| 30.0-34.9 |  | -0.240 | -0.184 |  |
|  |  | [-0.258,-0.223] | [-0.201,-0.167] |  |
| 35.0-39.9 |  | -0.311 | -0.246 |  |
|  |  | [-0.340,-0.283] | [-0.274,-0.218] |  |
| 40+ |  | -0.335 | -0.256 |  |
|  |  | [-0.375,-0.294] | [-0.295,-0.216] |  |
| Unknown |  | -0.159 | -0.079 |  |
|  |  | [-0.186,-0.133] | [-0.105,-0.054] |  |
|  |  |  |  |  |
| Maternal smoking |  | -0.324 | -0.160 |  |
|  |  | [-0.337,-0.311] | [-0.173,-0.146] |  |
|  |  |  |  |  |
| Male |  | -0.258 | -0.246 |  |
|  |  | [-0.266,-0.250] | [-0.255,-0.238] |  |
|  |  |  |  |  |
| Maternal age |  |  |  |  |
| <18 years |  |  | -0.266 |  |
|  |  |  | [-0.363,-0.168] |  |
| 18-24 years |  |  | -0.145 |  |
|  |  |  | [-0.162,-0.128] |  |
| 25-29 years |  |  | -0.050 |  |
|  |  |  | [-0.060,-0.040] |  |
| 30-34 years |  |  | Ref. |  |
|  |  |  |  |  |
| 35-39 years |  |  | 0.032 |  |
|  |  |  | [0.020,0.044] |  |
| 40+ years |  |  | 0.041 |  |
|  |  |  | [0.015,0.067] |  |
|  |  |  |  |  |
| Maternal partner = 1 |  |  | 0.084 |  |
|  |  |  | [0.069,0.099] |  |
|  |  |  |  |  |
| Maternal education |  |  |  |  |
| Lower secondary |  |  |  |  |
|  |  |  |  |  |
| Upper secondary |  |  | 0.190 |  |
|  |  |  | [0.176,0.203] |  |
| Tertiary |  |  | 0.448 |  |
|  |  |  | [0.433,0.463] |  |
|  |  |  |  |  |
| Maternal origin |  |  |  |  |
| Danish native |  |  | Ref. |  |
|  |  |  |  |  |
| 1st gen immigrant |  |  | -0.208 |  |
|  |  |  | [-0.223,-0.192] |  |
| 2nd gen immigrant |  |  | -0.251 |  |
|  |  |  | [-0.292,-0.210] |  |
| Unknown |  |  | -0.158 |  |
|  |  |  | [-0.241,-0.074] |  |
|  |  |  |  |  |
| Disposable income |  |  | 0.000 |  |
|  |  |  | [-0.000,0.000] |  |
| Income missing |  |  | 0.022 |  |
|  |  |  | [-0.041,0.085] |  |
|  |  |  |  |  |
| Age for grade |  |  |  |  |
| Young |  |  | 0.024 |  |
|  |  |  | [-0.008,0.056] |  |
| Old |  |  | -0.240 |  |
|  |  |  | [-0.259,-0.221] |  |
|  |  |  |  |  |
| Constant | 0.015 | 0.347 | 0.076 |  |
|  | [0.007,0.024] | [0.336,0.357] | [0.055,0.098] |  |
| Observations | 201240 | 201240 | 201240 |  |
| Standard errors clustered at maternal level. SC: Spontaneously conceived. ART: Assisted reproductive technology. IUI: Intrauterine insemination. ^a^ Adjusted for birth year. ^b^ Adjusted for birth year, multiple birth, parity, maternal body mass index (BMI), maternal smoking, and child sex. ^c^ Adjusted for birth year, multiple birth, parity, maternal body mass index (BMI), maternal smoking, maternal age, maternal relationship status, maternal education, maternal origin, maternal disposable income, child young (old) for grade and child sex. | | | | |

| Table A3: Linear model regressing third grade math score on mode of conception and covariates | | | | |
| --- | --- | --- | --- | --- |
|  | Baseline^a^ | Adjusted for birth related^b^ | Fully adjusted^c^ |  |
| ART | 0.092 | 0.042 | -0.043 |  |
|  | [0.063,0.121] | [0.012,0.072] | [-0.072,-0.014] |  |
| IUI | 0.145 | 0.099 | 0.016 |  |
|  | [0.103,0.187] | [0.057,0.140] | [-0.025,0.057] |  |
| Birth year |  |  |  |  |
| 2006 | Ref. | Ref. | Ref. |  |
|  |  |  |  |  |
| 2007 | 0.001 | -0.000 | -0.000 |  |
|  | [-0.011,0.014] | [-0.012,0.012] | [-0.012,0.011] |  |
| 2008 | 0.040 | 0.034 | 0.004 |  |
|  | [0.028,0.052] | [0.022,0.046] | [-0.008,0.016] |  |
| 2009 | 0.090 | 0.081 | -0.005 |  |
|  | [0.020,0.160] | [0.011,0.150] | [-0.081,0.071] |  |
|  |  |  |  |  |
| Multiple birth |  | -0.038 | -0.056 |  |
|  |  | [-0.066,-0.009] | [-0.083,-0.028] |  |
|  |  |  |  |  |
| Parity |  |  |  |  |
| 1 |  | Ref. | Ref. |  |
|  |  |  |  |  |
| 2 |  | -0.037 | -0.100 |  |
|  |  | [-0.048,-0.026] | [-0.111,-0.089] |  |
| 3 |  | -0.132 | -0.183 |  |
|  |  | [-0.148,-0.117] | [-0.199,-0.167] |  |
| 4+ |  | -0.348 | -0.291 |  |
|  |  | [-0.373,-0.322] | [-0.318,-0.265] |  |
| Unknown |  | -0.084 | -0.055 |  |
|  |  | [-0.116,-0.051] | [-0.087,-0.023] |  |
|  |  |  |  |  |
| Maternal BMI |  |  |  |  |
| <18.5 |  | -0.148 | -0.061 |  |
|  |  | [-0.168,-0.129] | [-0.079,-0.042] |  |
| 18.5-24.9 |  | Ref. | Ref. |  |
|  |  |  |  |  |
| 25.0-29.9 |  | -0.119 | -0.087 |  |
|  |  | [-0.132,-0.106] | [-0.100,-0.075] |  |
| 30.0-34.9 |  | -0.214 | -0.152 |  |
|  |  | [-0.234,-0.194] | [-0.171,-0.132] |  |
| 35.0-39.9 |  | -0.297 | -0.223 |  |
|  |  | [-0.329,-0.265] | [-0.254,-0.191] |  |
| 40+ |  | -0.300 | -0.205 |  |
|  |  | [-0.347,-0.252] | [-0.252,-0.159] |  |
| Unknown |  | -0.139 | -0.066 |  |
|  |  | [-0.169,-0.110] | [-0.095,-0.037] |  |
|  |  |  |  |  |
| Maternal smoking |  | -0.371 | -0.196 |  |
|  |  | [-0.385,-0.356] | [-0.211,-0.182] |  |
|  |  |  |  |  |
| Male |  | -0.017 | -0.003 |  |
|  |  | [-0.027,-0.007] | [-0.012,0.007] |  |
|  |  |  |  |  |
| Maternal age |  |  |  |  |
| <18 years |  |  | -0.229 |  |
|  |  |  | [-0.330,-0.128] |  |
| 18-24 years |  |  | -0.143 |  |
|  |  |  | [-0.162,-0.123] |  |
| 25-29 years |  |  | -0.048 |  |
|  |  |  | [-0.060,-0.036] |  |
| 30-34 years |  |  | Ref. |  |
|  |  |  |  |  |
| 35-39 years |  |  | 0.015 |  |
|  |  |  | [0.001,0.030] |  |
| 40+ years |  |  | -0.006 |  |
|  |  |  | [-0.036,0.025] |  |
|  |  |  |  |  |
| Maternal partner = 1 |  |  | 0.094 |  |
|  |  |  | [0.077,0.111] |  |
|  |  |  |  |  |
| Maternal education |  |  |  |  |
| Lower secondary |  |  | Ref. |  |
|  |  |  |  |  |
| Upper secondary |  |  | 0.198 |  |
|  |  |  | [0.183,0.213] |  |
| Tertiary |  |  | 0.480 |  |
|  |  |  | [0.463,0.497] |  |
|  |  |  |  |  |
| Maternal origin |  |  |  |  |
| Danish native |  |  | Ref. |  |
|  |  |  |  |  |
| 1st gen immigrant |  |  | -0.175 |  |
|  |  |  | [-0.192,-0.158] |  |
| 2nd gen immigrant |  |  | -0.238 |  |
|  |  |  | [-0.283,-0.192] |  |
| Unknown |  |  | -0.158 |  |
|  |  |  | [-0.254,-0.062] |  |
|  |  |  |  |  |
| Disposable income |  |  | 0.000 |  |
|  |  |  | [-0.000,0.001] |  |
| Income missing |  |  | 0.123 |  |
|  |  |  | [0.054,0.191] |  |
|  |  |  |  |  |
| Age for grade |  |  |  |  |
| Young |  |  | 0.057 |  |
|  |  |  | [0.019,0.094] |  |
| Old |  |  | -0.299 |  |
|  |  |  | [-0.321,-0.277] |  |
|  |  |  |  |  |
| Constant | 0.016 | 0.201 | -0.100 |  |
|  | [0.007,0.024] | [0.189,0.213] | [-0.125,-0.074] |  |
| Observations | 150653 | 150653 | 150653 |  |
| Standard errors clustered at maternal level. SC: Spontaneously conceived. ART: Assisted reproductive technology. IUI: Intrauterine insemination. ^a^ Adjusted for birth year. ^b^ Adjusted for birth year, multiple birth, parity, maternal body mass index (BMI), maternal smoking, and child sex. ^c^ Adjusted for birth year, multiple birth, parity, maternal body mass index (BMI), maternal smoking, maternal age, maternal relationship status, maternal education, maternal origin, maternal disposable income, child young (old) for grade and child sex. | | | | |

| Table A4: Linear model regressing second grade language comprehension and third grade math score on mode of conception and covariates by maternal education | | | | | |
| --- | --- | --- | --- | --- | --- |
|  | Grade 2,  < University^a^ | Grade 2,  University^a^ | Grade 3,  < University^a^ | Grade 3,  University^a^ |  |
| ART | -0.059 | -0.015 | -0.058 | -0.025 |  |
|  | [-0.095,-0.023] | [-0.047,0.017] | [-0.101,-0.015] | [-0.065,0.015] |  |
| IUI | 0.015 | 0.021 | 0.047 | -0.009 |  |
|  | [-0.031,0.061] | [-0.020,0.062] | [-0.012,0.106] | [-0.065,0.048] |  |
| IUI-ART | 0.074 | 0.036 | 0.105 | 0.017 |  |
|  | [-0.104,-0.037] | [-0.014,0.085] | [0.034,0.176] | [-0.050,0.083] |  |
| Birth year |  |  |  |  |  |
| 2006 | Ref. | Ref. | Ref. | Ref. |  |
|  |  |  |  |  |  |
| 2007 | 0.004 | -0.000 | 0.006 | -0.011 |  |
|  | [-0.011,0.019] | [-0.018,0.017] | [-0.009,0.020] | [-0.029,0.008] |  |
| 2008 | 0.015 | 0.000 | 0.004 | 0.004 |  |
|  | [0.000,0.030] | [-0.017,0.017] | [-0.012,0.019] | [-0.015,0.022] |  |
| 2009 | 0.016 | 0.001 | 0.004 | -0.015 |  |
|  | [0.001,0.032] | [-0.016,0.018] | [-0.103,0.111] | [-0.123,0.093] |  |
|  |  |  |  |  |  |
| Multiple birth | -0.070 | -0.096 | -0.042 | -0.072 |  |
|  | [-0.104,-0.037] | [-0.131,-0.062] | [-0.080,-0.004] | [-0.112,-0.032] |  |
|  |  |  |  |  |  |
| Parity |  |  |  |  |  |
| 1 | Ref. | Ref. | Ref. | Ref. |  |
|  |  |  |  |  |  |
| 2 | -0.160 | -0.118 | -0.112 | -0.086 |  |
|  | [-0.173,-0.147] | [-0.132,-0.104] | [-0.127,-0.097] | [-0.103,-0.068] |  |
| 3 | -0.250 | -0.175 | -0.202 | -0.157 |  |
|  | [-0.268,-0.231] | [-0.196,-0.155] | [-0.223,-0.182] | [-0.183,-0.131] |  |
| 4+ | -0.391 | -0.294 | -0.321 | -0.227 |  |
|  | [-0.420,-0.363] | [-0.340,-0.249] | [-0.351,-0.290] | [-0.279,-0.175] |  |
| Unknown | -0.087 | -0.064 | -0.068 | -0.035 |  |
|  | [-0.123,-0.051] | [-0.110,-0.017] | [-0.108,-0.028] | [-0.088,0.018] |  |
|  |  |  |  |  |  |
| Maternal BMI |  |  |  |  |  |
| <18.5 | -0.055 | -0.048 | -0.059 | -0.061 |  |
|  | [-0.075,-0.034] | [-0.075,-0.022] | [-0.082,-0.037] | [-0.092,-0.029] |  |
| 18.5-24.9 | Ref. | Ref. | Ref. | Ref. |  |
|  |  |  |  |  |  |
| 25.0-29.9 | -0.095 | -0.120 | -0.078 | -0.102 |  |
|  | [-0.109,-0.081] | [-0.137,-0.103] | [-0.094,-0.062] | [-0.123,-0.081] |  |
| 30.0-34.9 | -0.177 | -0.196 | -0.143 | -0.163 |  |
|  | [-0.198,-0.156] | [-0.225,-0.167] | [-0.167,-0.120] | [-0.198,-0.128] |  |
| 35.0-39.9 | -0.232 | -0.280 | -0.195 | -0.299 |  |
|  | [-0.265,-0.199] | [-0.330,-0.230] | [-0.231,-0.158] | [-0.361,-0.236] |  |
| 40+ | -0.252 | -0.255 | -0.190 | -0.247 |  |
|  | [-0.298,-0.207] | [-0.334,-0.177] | [-0.243,-0.138] | [-0.348,-0.146] |  |
| Unknown | -0.090 | -0.056 | -0.073 | -0.048 |  |
|  | [-0.123,-0.057] | [-0.097,-0.015] | [-0.109,-0.037] | [-0.096,-0.000] |  |
|  |  |  |  |  |  |
| Maternal smoking | -0.150 | -0.203 | -0.186 | -0.240 |  |
|  | [-0.165,-0.135] | [-0.234,-0.173] | [-0.202,-0.170] | [-0.275,-0.205] |  |
|  |  |  |  |  |  |
| Male | -0.265 | -0.217 | -0.013 | 0.014 |  |
|  | [-0.276,-0.254] | [-0.229,-0.205] | [-0.025,-0.001] | [-0.001,0.029] |  |
|  |  |  |  |  |  |
| Maternal age |  |  |  |  |  |
| <18 years | -0.276 | NA | -0.223 | NA |  |
|  | [-0.375,-0.178] |  | [-0.325,-0.122] |  |  |
| 18-24 years | -0.150 | -0.095 | -0.141 | -0.028 |  |
|  | [-0.169,-0.130] | [-0.215,0.025] | [-0.162,-0.120] | [-0.185,0.130] |  |
| 25-29 years | -0.044 | -0.059 | -0.038 | -0.062 |  |
|  | [-0.058,-0.030] | [-0.074,-0.043] | [-0.054,-0.022] | [-0.081,-0.042] |  |
| 30-34 years | Ref. | Ref. | Ref. | Ref. |  |
|  |  |  |  |  |  |
| 35-39 years | 0.051 | 0.009 | 0.030 | -0.001 |  |
|  | [0.032,0.070] | [-0.007,0.026] | [0.009,0.051] | [-0.022,0.019] |  |
| 40+ years | 0.058 | 0.017 | -0.004 | -0.010 |  |
|  | [0.020,0.096] | [-0.018,0.053] | [-0.047,0.039] | [-0.054,0.034] |  |
|  |  |  |  |  |  |
| Maternal partner = 1 | 0.092 | 0.051 | 0.088 | 0.110 |  |
|  | [0.074,0.110] | [0.023,0.080] | [0.069,0.108] | [0.075,0.145] |  |
|  |  |  |  |  |  |
| Maternal education |  |  |  |  |  |
| Lower secondary | Ref. |  | Ref. |  |  |
|  |  |  |  |  |  |
| Upper secondary | 0.190 |  | 0.199 |  |  |
|  | [0.176,0.204] |  | [0.183,0.214] |  |  |
|  |  |  |  |  |  |
| Maternal origin |  |  |  |  |  |
| Danish native | Ref. | Ref. | Ref. | Ref. |  |
|  |  |  |  |  |  |
| 1st gen immigrant | -0.203 | -0.217 | -0.158 | -0.214 |  |
|  | [-0.221,-0.185] | [-0.247,-0.187] | [-0.177,-0.139] | [-0.251,-0.177] |  |
| 2nd gen immigrant | -0.274 | -0.137 | -0.243 | -0.200 |  |
|  | [-0.321,-0.227] | [-0.215,-0.059] | [-0.293,-0.192] | [-0.305,-0.095] |  |
| Unknown | -0.146 |  | -0.141 |  |  |
|  | [-0.230,-0.061] |  | [-0.238,-0.044] |  |  |
|  |  |  |  |  |  |
| Disposable income | -0.000 | 0.000 | 0.001 | 0.000 |  |
|  | [-0.001,0.000] | [-0.000,0.000] | [0.000,0.001] | [-0.000,0.001] |  |
| Income missing | 0.020 | 1.385 | 0.121 | 1.150 |  |
|  | [-0.045,0.084] | [1.342,1.429] | [0.051,0.191] | [1.096,1.203] |  |
|  |  |  |  |  |  |
| Age for grade |  |  |  |  |  |
| Young | 0.035 | 0.014 | 0.057 | 0.058 |  |
|  | [-0.011,0.081] | [-0.030,0.058] | [0.005,0.109] | [0.004,0.112] |  |
| Old | -0.247 | -0.213 | -0.294 | -0.314 |  |
|  | [-0.270,-0.225] | [-0.248,-0.178] | [-0.320,-0.268] | [-0.359,-0.270] |  |
|  |  |  |  |  |  |
| Constant | 0.086 | 0.536 | -0.108 | 0.367 |  |
|  | [0.058,0.114] | [0.505,0.568] | [-0.138,-0.078] | [0.328,0.405] |  |
| Observations | 123810 | 77430 | 93356 | 57297 |  |
| Standard errors clustered at maternal level. SC: Spontaneously conceived. ART: Assisted reproductive technology. IUI: Intrauterine insemination. NA: No observation in category. ^a^ Adjusted for birth year, multiple birth, parity, maternal body mass index (BMI), maternal smoking, maternal age, maternal relationship status, maternal education, maternal origin, maternal disposable income, child young (old) for grade and child sex. | | | | | |

| Table A5: Linear model regressing second grade language comprehension score on mode of conception and covariates for singleton births | | | | |
| --- | --- | --- | --- | --- |
|  | Baseline^a^ | Adjusted for birth related^b^ | Fully adjusted^c^ |  |
| ART | 0.145 | 0.071 | -0.028 |  |
|  | [0.118,0.173] | [0.044,0.098] | [-0.055,-0.002] |  |
| IUI | 0.183 | 0.125 | 0.028 |  |
|  | [0.148,0.217] | [0.092,0.159] | [-0.005,0.061] |  |
| IUI-ART^d^ | 0.037 | 0.054 | 0.057 |  |
|  | [-0.006,0.080] | [0.012,0.096] | [0.015,0.098] |  |
| Birth year |  |  |  |  |
| 2006 | Ref. | Ref. | Ref. |  |
|  |  |  |  |  |
| 2007 | 0.004 | 0.002 | 0.004 |  |
|  | [-0.009,0.016] | [-0.010,0.014] | [-0.008,0.015] |  |
| 2008 | 0.007 | 0.007 | 0.009 |  |
|  | [-0.005,0.019] | [-0.004,0.019] | [-0.003,0.020] |  |
| 2009 | 0.045 | 0.033 | 0.010 |  |
|  | [0.033,0.058] | [0.021,0.045] | [-0.002,0.021] |  |
| Parity |  |  |  |  |
| 1 |  | Ref. | Ref. |  |
|  |  |  |  |  |
| 2 |  | -0.078 | -0.144 |  |
|  |  | [-0.087,-0.068] | [-0.153,-0.134] |  |
| 3 |  | -0.160 | -0.221 |  |
|  |  | [-0.173,-0.146] | [-0.235,-0.207] |  |
| 4+ |  | -0.405 | -0.359 |  |
|  |  | [-0.429,-0.381] | [-0.384,-0.335] |  |
| Unknown |  | -0.107 | -0.077 |  |
|  |  | [-0.137,-0.077] | [-0.106,-0.048] |  |
|  |  |  |  |  |
| Maternal BMI |  |  |  |  |
| <18.5 |  | -0.146 | -0.053 |  |
|  |  | [-0.163,-0.129] | [-0.069,-0.036] |  |
| 18.5-24.9 |  | Ref. | Ref. |  |
|  |  |  |  |  |
| 25.0-29.9 |  | -0.136 | -0.106 |  |
|  |  | [-0.147,-0.124] | [-0.117,-0.095] |  |
| 30.0-34.9 |  | -0.239 | -0.183 |  |
|  |  | [-0.257,-0.221] | [-0.200,-0.166] |  |
| 35.0-39.9 |  | -0.311 | -0.246 |  |
|  |  | [-0.340,-0.282] | [-0.275,-0.218] |  |
| 40+ |  | -0.339 | -0.261 |  |
|  |  | [-0.380,-0.298] | [-0.301,-0.221] |  |
| Unknown |  | -0.160 | -0.080 |  |
|  |  | [-0.187,-0.134] | [-0.106,-0.053] |  |
|  |  |  |  |  |
| Maternal smoking |  | -0.325 | -0.160 |  |
|  |  | [-0.339,-0.312] | [-0.174,-0.147] |  |
|  |  |  |  |  |
| Male |  | -0.259 | -0.248 |  |
|  |  | [-0.268,-0.251] | [-0.256,-0.239] |  |
|  |  |  |  |  |
| Maternal age |  |  |  |  |
| <18 years |  |  | -0.262 |  |
|  |  |  | [-0.360,-0.164] |  |
| 18-24 years |  |  | -0.144 |  |
|  |  |  | [-0.161,-0.126] |  |
| 25-29 years |  |  | -0.050 |  |
|  |  |  | [-0.061,-0.040] |  |
| 30-34 years |  |  | Ref. |  |
|  |  |  |  |  |
| 35-39 years |  |  | 0.031 |  |
|  |  |  | [0.019,0.044] |  |
| 40+ years |  |  | 0.041 |  |
|  |  |  | [0.014,0.067] |  |
|  |  |  |  |  |
| Maternal partner = 1 |  |  | 0.088 |  |
|  |  |  | [0.073,0.104] |  |
|  |  |  |  |  |
| Maternal education |  |  |  |  |
| Lower secondary |  |  | Ref. |  |
|  |  |  |  |  |
| Upper secondary |  |  | 0.190 |  |
|  |  |  | [0.176,0.204] |  |
| Tertiary |  |  | 0.449 |  |
|  |  |  | [0.434,0.463] |  |
|  |  |  |  |  |
| Maternal origin |  |  |  |  |
| Danish native |  |  | Ref. |  |
|  |  |  |  |  |
| 1st gen immigrant |  |  | -0.208 |  |
|  |  |  | [-0.224,-0.193] |  |
| 2nd gen immigrant |  |  | -0.258 |  |
|  |  |  | [-0.300,-0.216] |  |
| Unknown |  |  | -0.161 |  |
|  |  |  | [-0.246,-0.077] |  |
|  |  |  |  |  |
| Disposable income |  |  | 0.000 |  |
|  |  |  | [-0.000,0.000] |  |
| Income missing |  |  | 0.021 |  |
|  |  |  | [-0.042,0.085] |  |
|  |  |  |  |  |
| Age for grade |  |  |  |  |
| Young |  |  | 0.021 |  |
|  |  |  | [-0.011,0.054] |  |
| Old |  |  | -0.246 |  |
|  |  |  | [-0.265,-0.226] |  |
|  |  |  |  |  |
| Constant | 0.016 | 0.346 | 0.073 |  |
|  | [0.008,0.025] | [0.335,0.357] | [0.051,0.095] |  |
| Observations | 192661 | 192661 | 192661 |  |
| Standard errors clustered at maternal level. SC: Spontaneously conceived. ART: Assisted reproductive technology. IUI: Intrauterine insemination. ^a^ Adjusted for birth year. ^b^ Adjusted for birth year, multiple birth, parity, maternal body mass index (BMI), maternal smoking, and child sex. ^c^ Adjusted for birth year, multiple birth, parity, maternal body mass index (BMI), maternal smoking, maternal age, maternal relationship status, maternal education, maternal origin, maternal disposable income, child young (old) for grade and child sex. ^d^ Calculated separately. | | | | |

| Table A6: Linear model regressing third grade mathematics score on mode of conception and covariates for singleton births | | | | |
| --- | --- | --- | --- | --- |
|  | Baseline^a^ | Adjusted for birth related^b^ | Fully adjusted^c^ |  |
| ART | 0.114 | 0.051 | -0.043 |  |
|  | [0.081,0.148] | [0.017,0.084] | [-0.075,-0.010] |  |
| IUI | 0.147 | 0.096 | 0.005 |  |
|  | [0.101,0.193] | [0.051,0.142] | [-0.040,0.050] |  |
| IUI-ART^d^ | 0.033 | 0.046 | 0.048 |  |
|  | [-0.023,0.089] | [-0.010,0.101] | [-0.006,0.102] |  |
| Birth year |  |  |  |  |
| 2006 | Ref. | Ref. | Ref. |  |
|  |  |  |  |  |
| 2007 | 0.002 | 0.001 | 0.001 |  |
|  | [-0.010,0.015] | [-0.011,0.013] | [-0.011,0.013] |  |
| 2008 | 0.041 | 0.035 | 0.005 |  |
|  | [0.029,0.054] | [0.023,0.048] | [-0.007,0.017] |  |
| 2009 | 0.094 | 0.085 | -0.000 |  |
|  | [0.024,0.164] | [0.016,0.154] | [-0.076,0.076] |  |
| Parity |  |  |  |  |
| 1 |  | Ref. | Ref. |  |
|  |  |  |  |  |
| 2 |  | -0.034 | -0.098 |  |
|  |  | [-0.045,-0.023] | [-0.110,-0.087] |  |
| 3 |  | -0.130 | -0.183 |  |
|  |  | [-0.146,-0.114] | [-0.200,-0.167] |  |
| 4+ |  | -0.352 | -0.295 |  |
|  |  | [-0.378,-0.326] | [-0.322,-0.269] |  |
| Unknown |  | -0.081 | -0.052 |  |
|  |  | [-0.114,-0.047] | [-0.084,-0.019] |  |
|  |  |  |  |  |
| Maternal BMI |  |  |  |  |
| <18.5 |  | -0.150 | -0.060 |  |
|  |  | [-0.169,-0.130] | [-0.079,-0.041] |  |
| 18.5-24.9 |  | Ref. | Ref. |  |
|  |  |  |  |  |
| 25.0-29.9 |  | -0.119 | -0.087 |  |
|  |  | [-0.133,-0.106] | [-0.100,-0.075] |  |
| 30.0-34.9 |  | -0.214 | -0.151 |  |
|  |  | [-0.234,-0.194] | [-0.171,-0.132] |  |
| 35.0-39.9 |  | -0.304 | -0.230 |  |
|  |  | [-0.336,-0.271] | [-0.262,-0.198] |  |
| 40+ |  | -0.301 | -0.209 |  |
|  |  | [-0.349,-0.253] | [-0.256,-0.161] |  |
| Unknown |  | -0.142 | -0.067 |  |
|  |  | [-0.172,-0.112] | [-0.096,-0.038] |  |
|  |  |  |  |  |
| Maternal smoking |  | -0.371 | -0.196 |  |
|  |  | [-0.385,-0.356] | [-0.211,-0.181] |  |
|  |  |  |  |  |
| Male |  | -0.019 | -0.005 |  |
|  |  | [-0.029,-0.009] | [-0.015,0.005] |  |
|  |  |  |  |  |
| Maternal age |  |  |  |  |
| <18 years |  |  | -0.223 |  |
|  |  |  | [-0.323,-0.123] |  |
| 18-24 years |  |  | -0.142 |  |
|  |  |  | [-0.162,-0.123] |  |
| 25-29 years |  |  | -0.049 |  |
|  |  |  | [-0.061,-0.037] |  |
| 30-34 years |  |  | Ref. |  |
|  |  |  |  |  |
| 35-39 years |  |  | 0.017 |  |
|  |  |  | [0.002,0.032] |  |
| 40+ years |  |  | -0.009 |  |
|  |  |  | [-0.040,0.022] |  |
|  |  |  |  |  |
| Maternal partner = 1 |  |  | 0.093 |  |
|  |  |  | [0.076,0.110] |  |
|  |  |  |  |  |
| Maternal education |  |  |  |  |
| Lower secondary |  |  | Ref. |  |
|  |  |  |  |  |
| Upper secondary |  |  | 0.199 |  |
|  |  |  | [0.184,0.214] |  |
| Tertiary |  |  | 0.482 |  |
|  |  |  | [0.465,0.499] |  |
|  |  |  |  |  |
| Maternal origin |  |  |  |  |
| Danish native |  |  | Ref. |  |
|  |  |  |  |  |
| 1st gen immigrant |  |  | -0.176 |  |
|  |  |  | [-0.193,-0.159] |  |
| 2nd gen immigrant |  |  | -0.241 |  |
|  |  |  | [-0.287,-0.195] |  |
| Unknown |  |  | -0.184 |  |
|  |  |  | [-0.274,-0.094] |  |
|  |  |  |  |  |
| Disposable income |  |  | 0.000 |  |
|  |  |  | [-0.000,0.001] |  |
| Income missing |  |  | 0.126 |  |
|  |  |  | [0.057,0.194] |  |
|  |  |  |  |  |
| Age for grade |  |  |  |  |
| Young |  |  | 0.058 |  |
|  |  |  | [0.020,0.096] |  |
| Old |  |  | -0.304 |  |
|  |  |  | [-0.327,-0.281] |  |
|  |  |  |  |  |
| Constant | 0.016 | 0.200 | -0.099 |  |
|  | [0.007,0.025] | [0.188,0.212] | [-0.125,-0.073] |  |
| Observations | 144249 | 144249 | 144249 |  |
| Standard errors clustered at maternal level. SC: Spontaneously conceived. ART: Assisted reproductive technology. IUI: Intrauterine insemination. ^a^ Adjusted for birth year. ^b^ Adjusted for birth year, multiple birth, parity, maternal body mass index (BMI), maternal smoking, and child sex. ^c^ Adjusted for birth year, multiple birth, parity, maternal body mass index (BMI), maternal smoking, maternal age, maternal relationship status, maternal education, maternal origin, maternal disposable income, child young (old) for grade and child sex. ^d^ Calculated separately. | | | | |

| Table A7: Linear model regressing second grade language comprehension score on mode of conception and covariates for first births | | | | |
| --- | --- | --- | --- | --- |
|  | Baseline^a^ | Adjusted for birth related^b^ | Fully adjusted^c^ |  |
| ART | 0.057 | 0.031 | -0.057 |  |
|  | [0.026,0.088] | [0.000,0.062] | [-0.088,-0.026] |  |
| IUI | 0.133 | 0.118 | 0.025 |  |
|  | [0.094,0.172] | [0.080,0.157] | [-0.013,0.064] |  |
| IUI-ART^d^ | 0.075 | 0.087 | 0.082 |  |
|  | [0.027,0.124] | [0.039,0.135] | [0.035,0.129] |  |
| Birth year |  |  |  |  |
| 2006 | Ref. | Ref. | Ref. |  |
|  |  |  |  |  |
| 2007 | 0.001 | -0.001 | 0.003 |  |
|  | [-0.017,0.018] | [-0.018,0.016] | [-0.014,0.020] |  |
| 2008 | -0.006 | -0.008 | 0.003 |  |
|  | [-0.024,0.012] | [-0.026,0.009] | [-0.014,0.020] |  |
| 2009 | 0.035 | 0.025 | 0.013 |  |
|  | [0.016,0.053] | [0.007,0.043] | [-0.005,0.030] |  |
|  |  |  |  |  |
| Multiple births |  | -0.044 | -0.065 |  |
|  |  | [-0.079,-0.010] | [-0.099,-0.032] |  |
|  |  |  |  |  |
| Maternal BMI |  |  |  |  |
| <18.5 |  | -0.156 | -0.061 |  |
|  |  | [-0.182,-0.131] | [-0.086,-0.036] |  |
| 18.5-24.9 |  | Ref. | Ref. |  |
|  |  |  |  |  |
| 25.0-29.9 |  | -0.129 | -0.111 |  |
|  |  | [-0.146,-0.112] | [-0.127,-0.094] |  |
| 30.0-34.9 |  | -0.219 | -0.182 |  |
|  |  | [-0.245,-0.192] | [-0.208,-0.157] |  |
| 35.0-39.9 |  | -0.341 | -0.296 |  |
|  |  | [-0.387,-0.295] | [-0.342,-0.251] |  |
| 40+ |  | -0.333 | -0.276 |  |
|  |  | [-0.395,-0.271] | [-0.336,-0.215] |  |
| Unknown |  | -0.150 | -0.091 |  |
|  |  | [-0.195,-0.104] | [-0.135,-0.047] |  |
|  |  |  |  |  |
| Maternal smoking |  | -0.322 | -0.150 |  |
|  |  | [-0.341,-0.303] | [-0.170,-0.131] |  |
|  |  |  |  |  |
| Male |  | -0.246 | -0.235 |  |
|  |  | [-0.259,-0.234] | [-0.247,-0.223] |  |
|  |  |  |  |  |
| Maternal age |  |  |  |  |
| <18 years |  |  | -0.261 |  |
|  |  |  | [-0.365,-0.157] |  |
| 18-24 years |  |  | -0.129 |  |
|  |  |  | [-0.151,-0.107] |  |
| 25-29 years |  |  | -0.031 |  |
|  |  |  | [-0.045,-0.016] |  |
| 30-34 years |  |  | Ref. |  |
|  |  |  |  |  |
| 35-39 years |  |  | -0.002 |  |
|  |  |  | [-0.026,0.021] |  |
| 40+ years |  |  | -0.004 |  |
|  |  |  | [-0.054,0.045] |  |
|  |  |  |  |  |
| Maternal partner = 1 |  |  | 0.073 |  |
|  |  |  | [0.054,0.092] |  |
|  |  |  |  |  |
| Maternal education |  |  |  |  |
| Lower secondary |  |  | Ref. |  |
|  |  |  |  |  |
| Upper secondary |  |  | 0.203 |  |
|  |  |  | [0.184,0.223] |  |
| Tertiary |  |  | 0.445 |  |
|  |  |  | [0.423,0.466] |  |
|  |  |  |  |  |
| Maternal origin |  |  |  |  |
| Danish native |  |  | Ref. |  |
|  |  |  |  |  |
| 1st gen immigrant |  |  | -0.131 |  |
|  |  |  | [-0.154,-0.107] |  |
| 2nd gen immigrant |  |  | -0.280 |  |
|  |  |  | [-0.337,-0.223] |  |
| Unknown |  |  | -0.161 |  |
|  |  |  | [-0.312,-0.010] |  |
|  |  |  |  |  |
| Disposable income |  |  | 0.000 |  |
|  |  |  | [-0.000,0.000] |  |
| Income missing |  |  | -0.050 |  |
|  |  |  | [-0.138,0.039] |  |
|  |  |  |  |  |
| Age for grade |  |  |  |  |
| Young |  |  | 0.013 |  |
|  |  |  | [-0.041,0.066] |  |
| Old |  |  | -0.230 |  |
|  |  |  | [-0.258,-0.203] |  |
|  |  |  |  |  |
| Constant | 0.101 | 0.345 | 0.064 |  |
|  | [0.088,0.113] | [0.331,0.360] | [0.033,0.094] |  |
| Observations | 87612 | 87612 | 87612 |  |
| Standard errors clustered at maternal level. SC: Spontaneously conceived. ART: Assisted reproductive technology. IUI: Intrauterine insemination. ^a^ Adjusted for birth year. ^b^ Adjusted for birth year, multiple birth, parity, maternal body mass index (BMI), maternal smoking, and child sex. ^c^ Adjusted for birth year, multiple birth, parity, maternal body mass index (BMI), maternal smoking, maternal age, maternal relationship status, maternal education, maternal origin, maternal disposable income, child young (old) for grade and child sex. ^d^ Calculated separately. | | | | |

| Table A8: Linear model regressing third grade mathematics score on mode of conception and covariates for first births | | | | |
| --- | --- | --- | --- | --- |
|  | Baseline^a^ | Adjusted for birth related^b^ | Fully adjusted^c^ |  |
| ART | 0.059 | 0.023 | -0.067 |  |
|  | [0.022,0.096] | [-0.014,0.061] | [-0.104,-0.030] |  |
| IUI | 0.153 | 0.129 | 0.037 |  |
|  | [0.100,0.205] | [0.076,0.182] | [-0.014,0.089] |  |
| IUI-ART^d^ | 0.094 | 0.105 | 0.105 |  |
|  | [0.031,0.157] | [0.043,0.168] | [0.043,0.166] |  |
| Birth year |  |  |  |  |
| 2006 | Ref. | Ref. | Ref. |  |
|  |  |  |  |  |
| 2007 | 0.002 | 0.002 | 0.005 |  |
|  | [-0.016,0.021] | [-0.016,0.020] | [-0.012,0.023] |  |
| 2008 | 0.035 | 0.029 | 0.009 |  |
|  | [0.017,0.054] | [0.010,0.047] | [-0.009,0.027] |  |
| 2009 | 0.057 | 0.047 | -0.019 |  |
|  | [-0.073,0.187] | [-0.081,0.175] | [-0.160,0.122] |  |
|  |  |  |  |  |
| Multiple births |  | -0.012 | -0.039 |  |
|  |  | [-0.053,0.030] | [-0.079,0.000] |  |
|  |  |  |  |  |
| Maternal BMI |  |  |  |  |
| <18.5 |  | -0.118 | -0.094 |  |
|  |  | [-0.137,-0.098] | [-0.113,-0.075] |  |
| 18.5-24.9 |  | Ref. | Ref. |  |
|  |  |  |  |  |
| 25.0-29.9 |  | -0.209 | -0.163 |  |
|  |  | [-0.240,-0.179] | [-0.193,-0.134] |  |
| 30.0-34.9 |  | -0.277 | -0.220 |  |
|  |  | [-0.329,-0.224] | [-0.272,-0.168] |  |
| 35.0-39.9 |  | -0.297 | -0.217 |  |
|  |  | [-0.372,-0.222] | [-0.292,-0.143] |  |
| 40+ |  | -0.130 | -0.072 |  |
|  |  | [-0.183,-0.077] | [-0.123,-0.021] |  |
| Unknown |  | -0.118 | -0.094 |  |
|  |  | [-0.137,-0.098] | [-0.113,-0.075] |  |
|  |  |  |  |  |
| Maternal smoking |  | -0.378 | -0.189 |  |
|  |  | [-0.400,-0.356] | [-0.211,-0.167] |  |
|  |  |  |  |  |
| Male |  | -0.007 | 0.005 |  |
|  |  | [-0.022,0.008] | [-0.009,0.019] |  |
|  |  |  |  |  |
| Maternal age |  |  |  |  |
| <18 years |  |  | -0.230 |  |
|  |  |  | [-0.338,-0.121] |  |
| 18-24 years |  |  | -0.146 |  |
|  |  |  | [-0.172,-0.120] |  |
| 25-29 years |  |  | -0.038 |  |
|  |  |  | [-0.056,-0.020] |  |
| 30-34 years |  |  | Ref. |  |
|  |  |  |  |  |
| 35-39 years |  |  | -0.026 |  |
|  |  |  | [-0.055,0.003] |  |
| 40+ years |  |  | -0.034 |  |
|  |  |  | [-0.096,0.029] |  |
|  |  |  |  |  |
| Maternal partner = 1 |  |  | 0.080 |  |
|  |  |  | [0.058,0.102] |  |
|  |  |  |  |  |
| Maternal education |  |  |  |  |
| Lower secondary |  |  | Ref. |  |
|  |  |  |  |  |
| Upper secondary |  |  | 0.230 |  |
|  |  |  | [0.207,0.253] |  |
| Tertiary |  |  | 0.485 |  |
|  |  |  | [0.459,0.511] |  |
|  |  |  |  |  |
| Maternal origin |  |  |  |  |
| Danish native |  |  | Ref. |  |
|  |  |  |  |  |
| 1st gen immigrant |  |  | -0.091 |  |
|  |  |  | [-0.118,-0.063] |  |
| 2nd gen immigrant |  |  | -0.265 |  |
|  |  |  | [-0.333,-0.197] |  |
| Unknown |  |  | -0.142 |  |
|  |  |  | [-0.337,0.054] |  |
|  |  |  |  |  |
| Disposable income |  |  | 0.001 |  |
|  |  |  | [0.000,0.002] |  |
| Income missing |  |  | 0.111 |  |
|  |  |  | [0.015,0.206] |  |
|  |  |  |  |  |
| Age for grade |  |  |  |  |
| Young |  |  | 0.046 |  |
|  |  |  | [-0.018,0.111] |  |
| Old |  |  | -0.284 |  |
|  |  |  | [-0.318,-0.250] |  |
|  |  |  |  |  |
| Constant | 0.072 | 0.197 | -0.130 |  |
|  | [0.060,0.085] | [0.181,0.212] | [-0.166,-0.093] |  |
| Observations | 65313 | 65313 | 65313 |  |
| Standard errors clustered at maternal level. SC: Spontaneously conceived. ART: Assisted reproductive technology. IUI: Intrauterine insemination. ^a^ Adjusted for birth year. ^b^ Adjusted for birth year, multiple birth, parity, maternal body mass index (BMI), maternal smoking, and child sex. ^c^ Adjusted for birth year, multiple birth, parity, maternal body mass index (BMI), maternal smoking, maternal age, maternal relationship status, maternal education, maternal origin, maternal disposable income, child young (old) for grade and child sex. ^d^ Calculated separately. | | | | |

| Table A9: Linear model regressing second grade language comprehension score on mode of conception and covariates for first births | | | | |
| --- | --- | --- | --- | --- |
|  | Baseline^a^ | Adjusted for birth related^b^ | Fully adjusted^c^ |  |
| ART | 0.120 | 0.070 | -0.026 |  |
|  | [0.094,0.145] | [0.044,0.096] | [-0.052,-0.000] |  |
| IUI | 0.158 | 0.117 | 0.021 |  |
|  | [0.125,0.190] | [0.084,0.149] | [-0.010,0.053] |  |
| IUI-ART^d^ | 0.038 | 0.046 | 0.047 |  |
|  | [-0.003,0.079] | [0.007,0.086] | [0.008,0.086] |  |
| Birth year |  |  |  |  |
| 2007 | Ref. | Ref. | Ref. |  |
|  |  |  |  |  |
| 2008 | 0.005 | 0.006 | 0.007 |  |
|  | [-0.007,0.017] | [-0.006,0.018] | [-0.004,0.018] |  |
| 2009 | 0.043 | 0.033 | 0.009 |  |
|  | [0.031,0.055] | [0.021,0.044] | [-0.003,0.021] |  |
| Parity |  |  |  | |
| 1 | Ref. | Ref. | Ref. | |
|  |  |  |  | |
| 2 | -0.077 | -0.143 | -0.077 | |
|  | [-0.088,-0.066] | [-0.154,-0.132] | [-0.088,-0.066] | |
| 3 | -0.151 | -0.213 | -0.151 | |
|  | [-0.167,-0.136] | [-0.229,-0.197] | [-0.167,-0.136] | |
| 4+ | -0.399 | -0.356 | -0.399 | |
|  | [-0.426,-0.372] | [-0.383,-0.328] | [-0.426,-0.372] | |
| Unknown | -0.112 | -0.082 | -0.112 | |
|  | [-0.145,-0.079] | [-0.115,-0.050] | [-0.145,-0.079] | |
|  |  |  |  |  |
| Multiple births |  | -0.077 | -0.088 |  |
|  |  | [-0.106,-0.048] | [-0.117,-0.060] |  |
|  |  |  |  |  |
| Maternal BMI |  |  |  |  |
| <18.5 |  | -0.149 | -0.058 |  |
|  |  | [-0.168,-0.129] | [-0.077,-0.039] |  |
| 18.5-24.9 |  | Ref. | Ref. |  |
|  |  |  |  |  |
| 25.0-29.9 |  | -0.130 | -0.100 |  |
|  |  | [-0.143,-0.117] | [-0.113,-0.088] |  |
| 30.0-34.9 |  | -0.240 | -0.186 |  |
|  |  | [-0.260,-0.220] | [-0.206,-0.167] |  |
| 35.0-39.9 |  | -0.318 | -0.256 |  |
|  |  | [-0.350,-0.285] | [-0.288,-0.225] |  |
| 40+ |  | -0.327 | -0.250 |  |
|  |  | [-0.373,-0.281] | [-0.295,-0.205] |  |
| Unknown |  | -0.158 | -0.070 |  |
|  |  | [-0.190,-0.125] | [-0.101,-0.039] |  |
|  |  |  |  |  |
| Maternal smoking |  | -0.316 | -0.150 |  |
|  |  | [-0.331,-0.300] | [-0.166,-0.134] |  |
|  |  |  |  |  |
| Male |  | -0.258 | -0.249 |  |
|  |  | [-0.268,-0.249] | [-0.259,-0.240] |  |
|  |  |  |  |  |
| Maternal age |  |  |  |  |
| <18 years |  |  | -0.294 |  |
|  |  |  | [-0.412,-0.176] |  |
| 18-24 years |  |  | -0.136 |  |
|  |  |  | [-0.156,-0.116] |  |
| 25-29 years |  |  | -0.043 |  |
|  |  |  | [-0.055,-0.031] |  |
| 30-34 years |  |  | Ref. |  |
|  |  |  |  |  |
| 35-39 years |  |  | 0.034 |  |
|  |  |  | [0.019,0.048] |  |
| 40+ years |  |  | 0.035 |  |
|  |  |  | [0.005,0.065] |  |
|  |  |  |  |  |
| Maternal partner = 1 |  |  | 0.085 |  |
|  |  |  | [0.067,0.102] |  |
|  |  |  |  |  |
| Maternal education |  |  |  |  |
| Lower secondary |  |  | Ref. |  |
|  |  |  |  |  |
| Upper secondary |  |  | 0.187 |  |
|  |  |  | [0.171,0.202] |  |
| Tertiary |  |  | 0.447 |  |
|  |  |  | [0.431,0.464] |  |
|  |  |  |  |  |
| Maternal origin |  |  |  |  |
| Danish native |  |  | Ref. |  |
|  |  |  |  |  |
| 1st gen immigrant |  |  | -0.197 |  |
|  |  |  | [-0.215,-0.180] |  |
| 2nd gen immigrant |  |  | -0.266 |  |
|  |  |  | [-0.313,-0.220] |  |
| Unknown |  |  | -0.154 |  |
|  |  |  | [-0.252,-0.057] |  |
|  |  |  |  |  |
| Disposable income |  |  | 0.000 |  |
|  |  |  | [-0.000,0.000] |  |
| Income missing |  |  | -0.032 |  |
|  |  |  | [-0.105,0.041] |  |
|  |  |  |  |  |
| Age for grade |  |  |  |  |
| Young |  |  | 0.027 |  |
|  |  |  | [-0.009,0.064] |  |
| Old |  |  | -0.229 |  |
|  |  |  | [-0.253,-0.206] |  |
|  |  |  |  |  |
| Constant | 0.018 | 0.343 | 0.073 |  |
|  | [0.009,0.026] | [0.331,0.354] | [0.048,0.097] |  |
| Observations | 148521 | 148521 | 148521 |  |
| Standard errors clustered at maternal level. SC: Spontaneously conceived. ART: Assisted reproductive technology. IUI: Intrauterine insemination. ^a^ Adjusted for birth year. ^b^ Adjusted for birth year, multiple birth, parity, maternal body mass index (BMI), maternal smoking, and child sex. ^c^ Adjusted for birth year, multiple birth, parity, maternal body mass index (BMI), maternal smoking, maternal age, maternal relationship status, maternal education, maternal origin, maternal disposable income, child young (old) for grade and child sex. ^d^ Calculated separately. | | | | |

| Table A10: Linear model regressing third grade mathematics score on mode of conception and covariates for first births | | | | |
| --- | --- | --- | --- | --- |
|  | Baseline^a^ | Adjusted for birth related^b^ | Fully adjusted^c^ |  |
| ART | 0.105 | 0.060 | -0.033 |  |
|  | [0.073,0.137] | [0.027,0.093] | [-0.065,-0.001] |  |
| IUI | 0.139 | 0.096 | 0.005 |  |
|  | [0.094,0.183] | [0.051,0.141] | [-0.038,0.049] |  |
| IUI-ART^d^ | 0.034 | 0.036 | 0.039 |  |
|  | [-0.021,0.088] | [-0.018,0.090] | [-0.014,0.091] |  |
| Birth year |  |  |  |  |
| 2007 | Ref. | Ref. | Ref. |  |
|  |  |  |  |  |
| 2008 | 0.039 | 0.034 | 0.005 |  |
|  | [0.027,0.051] | [0.022,0.046] | [-0.007,0.017] |  |
| 2009 | 0.089 | 0.081 | -0.027 |  |
|  | [0.019,0.159] | [0.012,0.150] | [-0.107,0.053] |  |
| Parity |  |  |  | |
| 1 | Ref. | Ref. | Ref. | |
|  |  |  |  | |
| 2 | -0.037 | -0.102 | -0.037 | |
|  | [-0.051,-0.023] | [-0.115,-0.088] | [-0.051,-0.023] | |
| 3 | -0.130 | -0.186 | -0.130 | |
|  | [-0.150,-0.111] | [-0.207,-0.166] | [-0.150,-0.111] | |
| 4+ | -0.330 | -0.278 | -0.330 | |
|  | [-0.362,-0.299] | [-0.310,-0.246] | [-0.362,-0.299] | |
| Unknown | -0.100 | -0.078 | -0.100 | |
|  | [-0.140,-0.061] | [-0.117,-0.039] | [-0.140,-0.061] | |
|  |  |  |  |  |
| Multiple births |  | -0.053 | -0.067 |  |
|  |  | [-0.089,-0.017] | [-0.102,-0.032] |  |
|  |  |  |  |  |
| Maternal BMI |  |  |  |  |
| <18.5 |  | -0.146 | -0.063 |  |
|  |  | [-0.170,-0.123] | [-0.085,-0.040] |  |
| 18.5-24.9 |  | Ref. | Ref. |  |
|  |  |  |  |  |
| 25.0-29.9 |  | -0.112 | -0.080 |  |
|  |  | [-0.128,-0.096] | [-0.096,-0.065] |  |
| 30.0-34.9 |  | -0.210 | -0.151 |  |
|  |  | [-0.234,-0.186] | [-0.175,-0.127] |  |
| 35.0-39.9 |  | -0.313 | -0.242 |  |
|  |  | [-0.352,-0.273] | [-0.281,-0.204] |  |
| 40+ |  | -0.275 | -0.179 |  |
|  |  | [-0.333,-0.217] | [-0.237,-0.122] |  |
| Unknown |  | -0.125 | -0.050 |  |
|  |  | [-0.163,-0.087] | [-0.087,-0.013] |  |
|  |  |  |  |  |
| Maternal smoking |  | -0.372 | -0.195 |  |
|  |  | [-0.390,-0.354] | [-0.214,-0.177] |  |
|  |  |  |  |  |
| Male |  | -0.015 | -0.006 |  |
|  |  | [-0.027,-0.003] | [-0.018,0.005] |  |
|  |  |  |  |  |
| Maternal age |  |  |  |  |
| <18 years |  |  | -0.183 |  |
|  |  |  | [-0.305,-0.060] |  |
| 18-24 years |  |  | -0.132 |  |
|  |  |  | [-0.156,-0.109] |  |
| 25-29 years |  |  | -0.040 |  |
|  |  |  | [-0.055,-0.025] |  |
| 30-34 years |  |  | Ref. |  |
|  |  |  |  |  |
| 35-39 years |  |  | 0.023 |  |
|  |  |  | [0.005,0.041] |  |
| 40+ years |  |  | -0.010 |  |
|  |  |  | [-0.048,0.027] |  |
|  |  |  |  |  |
| Maternal partner = 1 |  |  | 0.104 |  |
|  |  |  | [0.083,0.125] |  |
|  |  |  |  |  |
| Maternal education |  |  |  |  |
| Lower secondary |  |  | Ref. |  |
|  |  |  |  |  |
| Upper secondary |  |  | 0.200 |  |
|  |  |  | [0.181,0.218] |  |
| Tertiary |  |  | 0.483 |  |
|  |  |  | [0.463,0.504] |  |
|  |  |  |  |  |
| Maternal origin |  |  |  |  |
| Danish native |  |  | Ref. |  |
|  |  |  |  |  |
| 1st gen immigrant |  |  | -0.158 |  |
|  |  |  | [-0.179,-0.138] |  |
| 2nd gen immigrant |  |  | -0.226 |  |
|  |  |  | [-0.281,-0.172] |  |
| Unknown |  |  | -0.093 |  |
|  |  |  | [-0.212,0.026] |  |
|  |  |  |  |  |
| Disposable income |  |  | 0.000 |  |
|  |  |  | [-0.000,0.001] |  |
| Income missing |  |  | 0.088 |  |
|  |  |  | [0.006,0.170] |  |
|  |  |  |  |  |
| Age for grade |  |  |  |  |
| Young |  |  | 0.078 |  |
|  |  |  | [0.033,0.123] |  |
| Old |  |  | -0.295 |  |
|  |  |  | [-0.327,-0.263] |  |
|  |  |  |  |  |
| Constant | 0.017 | 0.197 | -0.116 |  |
|  | [0.008,0.026] | [0.184,0.211] | [-0.146,-0.086] |  |
| Observations | 98443 | 98443 | 98443 |  |
| Standard errors clustered at maternal level. SC: Spontaneously conceived. ART: Assisted reproductive technology. IUI: Intrauterine insemination. ^a^ Adjusted for birth year. ^b^ Adjusted for birth year, multiple birth, parity, maternal body mass index (BMI), maternal smoking, and child sex. ^c^ Adjusted for birth year, multiple birth, parity, maternal body mass index (BMI), maternal smoking, maternal age, maternal relationship status, maternal education, maternal origin, maternal disposable income, child young (old) for grade and child sex. ^d^ Calculated separately. | | | | |
