# Supplementary material for: The association between type of conception through medically assisted reproduction and childhood cognition: a Danish population-wide cohort study
Source: Eur J Public Health. 2023 Jul 22;33(6):1020–6. doi: 10.1093/eurpub/ckad123 (PMC10710341; doi:10.1093/eurpub/ckad123)
Supplement: ckad123_Supplementary_Data [file ckad123_supplementary_data.zip › ckad123_Supplementary_Data/ejph-2023-03-om-0148-File006.docx]

NAME: 00master.do

*******************************************************************************

*******************************************************************************

** MASTER FILE

** 11-26-2022

*******************************************************************************

*******************************************************************************

global dorg "ADMIN DATA"

global data "WORK FOLDER"

cd "IVF LOCATION"

import sas using t_grund.sas7bdat, clear

cd "$data"

save IVF2.dta, replace

do 01build_sample.do

do 02build_covar.do

do 03build_testdata.do

do 04estimate.do

NAME: 01build_sample.do

forvalue t = 2006/2009{

if `t' ==2006 use $dorg/mfr2006, clear

else append using $dorg/mfr`t'

}

rename cpr_moder pnr

keep if pnr !=""

bysort pnr: gen help =_n

keep if levende_eller_doedfoedt!="Dødfødt"

tempfile mfr

save `mfr'

sum help

local q = r(max)

tempfil ivf

forvalue t=1/`q'{

use `mfr' if help==`t'

merge 1:m pnr using $data/data/IVF2.dta, keep(1 3) nogen

replace C_B ="" if inlist(C_B,"SPONTAN") | V_GRAVID == "Aflyst" | V_PROEVE_TYPE =="Behandling afbrudt" | B_BEHANDLING_AFBRUDT==1

gen start_date = D_TRANSFERERING_DATO

replace start_date = D_IUI_DATO if start_date ==.

gen diff = foedselsdato-start_date

gen ivf = diff < 365.2425*10/12 & diff > 0

bysort pnr (ivf): keep if _n==_N

if `t' > 1 append using `ivf'

save `ivf', replace

}

gen IUI = C_B =="IUI" & ivf==1

gen ART = !inlist(C_B,"","SPONTAN") & IUI ==0 & ivf==1

replace C_B = "" if ivf==0

replace C_B = "OOCYT" if inlist(C_B,"OOCYT_DONOR","DISTRI")

replace C_B = "FER" if C_B == "ALM+FER"

replace C_B = "IVF/ICSI" if C_B =="ALM"

drop D_START

save $data/data/ivf_sample, replace

NAME: 02build_covar.do

tempfile covar

forvalue t =2005/2008{

local n = `t'-1

local m = `t'+1

use $data/data/ivf_sample if year(foedselsdato)==`m', clear

destring pnr, replace force

keep if pnr !=.

merge m:1 pnr using e:/data/rawdata/705830/udda`t', ///

keepus(hfaudd) keep(1 3) nogen

merge m:1 pnr using e:/data/rawdata/705830/bef12_`t', ///

keepus(ie_type e_faelle_id civst antpersh antboernh) keep(1 3) nogen

rename ie_type IE_TYPE

merge m:1 pnr using e:/data/rawdata/705830/bef12_`n', ///

keepus(ie_type ) keep(1 3) nogen

replace IE_TYPE = ie_type if IE_TYPE ==.

drop ie_type

merge m:1 pnr using e:/data/rawdata/705830/bef12_`m', ///

keepus( ie_type ) keep(1 3) nogen

replace IE_TYPE = ie_type if IE_TYPE ==.

drop ie_type

rename IE_TYPE ie_type

merge m:m pnr using e:/data/rawdata/705830/ind`t', ///a few income observations have double observations, but does not affect dispon_13

keepus(dispon_13) keep(1 3) nogen

bysort cpr_barn: keep if _n ==1 // correct for m:m merge at income

cap rename *`t' *

if `t' > 2005 append using `covar'

save `covar', replace

}

gen start=string(hfaudd,"%04.0f")

merge m:1 start using "\\SRVFSENAS1\data\Formater\SAS formater i Danmarks Statistik\STATA_datasaet\Disced\c_audd_level_l1l4_k.dta" , keep(1 3) nogen

destring AUDD, gen(isced)

drop AUDD start

gen level = 1 if isced < 3 | isced ==. | isced ==9

replace level = 2 if inlist(isced,3,4)

replace level = 3 if level ==.

rename dispon_13 disposable_income

gen partner = e_faelle_id !="" | civst==("G","P") | (antpersh-antboernh ==2)

save $data/data/ivf_covar.dta, replace

NAME: 03build_testdata.do

use "$dorg\nationale_testdata.dta" ///

if testtype =="obligatorisk", clear

**Keep relevant tests

replace klassetrin =0 if klassetrin ==.

bysort pnr skoleaar: egen kl = max(klassetrin)

gen disqualify =1

foreach x in 2 3 4 6 8{

replace disqualify = 0 if kl ==`x' & !regexm(fag,`"`x'"')

}

gen subject_matter =""

foreach x in Biologi Dansk/læsning Engelsk Fysik/kemi Geografi Matematik{

replace subject_matter = "`x'" if regexm(fag,"`x'")

}

gen subject_grade = .

forvalue t=2/8{

replace subject_grade= `t' if regexm(fag,"`t'")

}

drop if disqualify ==0

bysort pnr: gen help =_N

tab help

bysort pnr skoleaar: gen help1 =_N

tab help1

**keep relevant variables

keep pnr skoleaar fag klassetrin testtid theta* instnr subject_matter subject_grade

destring pnr, replace force

drop if pnr ==.

**standardize variables from logit scale across domains

foreach x in 1 2 3{

bysort subject_matter subject_grade skoleaar: egen mean = mean(theta_p`x')

bysort subject_matter subject_grade skoleaar: egen sd = sd(theta_p`x')

gen ztheta`x' = (theta_p`x'-mean)/sd

drop sd mean

}

*generate joint score

gen z_prelim = (ztheta1+ztheta2+ztheta3)/3

bysort skoleaar fag: egen mean = mean(z_prelim)

bysort skoleaar fag: egen sd = sd(z_prelim)

gen z_score = (z_prelim-mean)/sd

drop z_prelim mean sd

gen year = substr(testtid,1,4)

gen month= substr(testtid,6,2)

gen day = substr(testtid,9,2)

destring year, replace

destring month, replace

destring day, replace

drop theta* ztheta*

rename pnr pnr_child

rename fag subject

rename testtid testtime

rename instnr school_id

rename klassetrin grade

save $data\test_data.dta, replace

NAME: 04estimate.do

global dorg "e:/data/rawdata/705830"

global data "Y:\Data\Workdata\705830\pf\IVF_edu\"

eststo clear

use pnr handdto indud using "E:\Data\rawdata\705830\vnds2021.dta", clear

bysort pnr (hand): keep if _n==_N

keep if indud==2

drop indud

tempfile migrant

save `migrant'

use $data/data/ivf_covar.dta, clear

rename pnr mor_id

destring cpr_barn, gen(pnr_child) force

keep if pnr_child !=.

replace ivf =0 if ART ==0 & IUI==0

tab ivf

merge 1:m pnr_child using $data\data\test_data.dta, keep(1 3)

keep if subject_grade < 4 | subject_grade==.

gen byear = year(foedselsdato)

**remove children who migrate/die before age 9

rename pnr_child pnr

merge m:1 pnr using $dorg/dod2020, keep(1 3) nogen

drop if alder_haend < 9

merge m:1 pnr using `migrant', keep(1 3) nogen

keep if (handdto-foedselsdato)/365.2425 >=9

destring apgarscore_efter5minutter, replace

gen twin_plus = flerfoldsfoedsel_beregnet >1

label var twin_plus "Multiple birth"

replace vaegt_b =. if vaegt_b < 450 | vaegt_b ==9999

recode vaegt_b (450/1499=1 "450-1499 grams") (1500/2499=2 "1500-2499 grams") ///

(2500/3499=3 "2500-3499 grams") (3500/4499=4 "3500-4499 grams") ///

(4500/9000=5 "4500+ grams") (.=6 "Unknown") , gen(weight)

label var weight "Birthweight"

recode alder_moder (0/17 =1 "<18 years") (18/24 =2 "18-24 years") ///

(25/29 = 3 "25-29 years") (30/34 = 4 "30-34 years") ///

(35/39 = 5 "35-39 years") (40/61 = 6 "40+ years"), ///

gen(maternal_age)

label var maternal_age "Maternal age"

destring paritet, replace force

recode paritet (. = 5 "Unknown") (1=1 "Parity 1") (2=2 "2") (3=3 "3") (4/. = 4 "4+"), ///

gen(parity)

label var parity "Parity"

destring ryg, replace force

recode ryg (1/98 =1 "Smoker") (else = 0 "Non-smoker"), gen(smoker)

label var smoker "Smoked during pregnancy"

label var parity "Parity"

*order parity

bysort mor_id byear: egen helpX= min(parity)

replace parity = helpX if helpX != parity

drop helpX

**correct erro in coding

replace bmi_moder = bmi_moder/10 if bmi_moder > 100

replace vaegt_moder = vaegt_moder /100 if vaegt_moder > 3000

replace vaegt_moder = vaegt_moder /10 if vaegt_moder > 1000

replace hoejde_moder = hoejde_moder /10 if hoejde_moder > 1000

replace hoejde_moder = hoejde_moder +100 if hoejde_moder < 100

gen bmi2= vaegt_moder/((hoejde_moder/100)^2)

replace bmi_moder = bmi2 if bmi2 < bmi_moder & bmi2 < 55

recode bmi_moder (0/18.499 = 1 " 18.5") (18.5/24.999 = 2 "18.5-24.9") ///

(25/29.999 = 3 "25.0-29.9") (30.0/34.999 = 4 "30.0-34.9") ///

(35/39.999 = 5 "35.0-39.9") (40/55 = 6 "40+") ///

(0 55.001/99 . = 99 "Unknown"), gen(maternal_bmi)

label var maternal_bmi "Maternal BMI"

rename ie_ty ie

recode ie (1=1 "Native Dane") (2=2 "1st G Migrant") (3=3 "2nd G Migrant") (.=4 "Unknown"), gen(ie_type)

drop ie

label var ie_type "Migration background"

gen disp_mis = dispo ==.

label var disp_mis "Income missing"

replace dispo = 0 if dispo ==.

replace dispo = dispo/7450

label var dispo "Disp income in €1K"

label var level "Maternal level of Education"

gen young = year-byear <= 6+subject_grade

label var young "Young for grade"

gen old = year-byear > 6+subject_grade+1

label var old "Old for grade"

gen male = koen=="M"

label var male "Male"

label var ivf "MAR"

label var byear "Birth year"

label var partner "In relationship"

**IS IVF correlated with not in sample

bysort pnr: gen mark= _n ==1

gen in_sample = (z_score !=. & subject_grade < 4) if mark==1

tab ivf if mark==1

drop mark

**ABSENT FROM OUTCOME

reg in_sample i.ivf i.byear

eststo e1

margins i.ivf

reg in_sample i.ivf i.byear twin_plus i.b4.maternal_age i.parity i.b2.maternal_bmi smoker partner i.level i.ie_type dispo disp_ male

eststo e2

margins ivf

esttab e* using $data/tables/TableA1.rtf, plain par b(3) ci(3) label replace

drop if subject_grade ==.

gen TREATED = "NC"

replace TREATED = "ART" if ART==1

replace TREATED = "IUI" if IUI==1

gen NC = ART==0 & IUI ==0

tab subject_grade TREATED, col

tab male TREATED, col

tab byear TREATED, col

tab weight TREATED, col

tab twin_plus TREATED, col

tab maternal_age TREATED, col

tab parity TREATED, col

tab maternal_bmi TREATED, col

tab smoker TREATED, col

tab partner TREATED, col

tab level TREATED, col

tab ie_type TREATED, col

tab young TREATED, col

tab old TREATED, col

table () (TREATED), stat(mean dispo) stat(sd dispo)

encode TREATED, gen(treated)

reg z_score i.byear i.b3.treated if subject_grade==2, cl(mor_id)

eststo e1

margins treated

reg z_score i.byear i.b3.treated twin_plus i.parity i.b2.maternal_bmi smoker male if subject_grade==2, cl(mor_id)

eststo e2

margins treated

reg z_score i.byear i.b3.treated twin_plus i.b4.maternal_age i.parity i.b2.maternal_bmi smoker partner i.level i.ie_type dispo disp_ young old male if subject_grade==2, cl(mor_id)

eststo e3

margins treated

esttab e* using $data/tables/grade2_both.rtf, plain par b(3) ci(3) label replace

gen art = treated ==1

gen iui = treated ==2

reg z_score i.byear art iui twin_plus i.b4.maternal_age i.parity i.b2.maternal_bmi smoker partner i.level i.ie_type dispo disp_ young old male if subject_grade==2, cl(mor_id)

reg z_score i.byear i.b3.treated if subject_grade==3, cl(mor_id)

eststo e1

margins treated

reg z_score i.byear i.b3.treated twin_plus i.parity i.b2.maternal_bmi smoker male if subject_grade==3, cl(mor_id)

eststo e2

margins treated

reg z_score i.byear i.b3.treated twin_plus i.b4.maternal_age i.parity i.b2.maternal_bmi smoker partner i.level i.ie_type dispo disp_ young old male if subject_grade==3, cl(mor_id)

eststo e3

margins treated

esttab e* using $data/tables/grade3_both.rtf, plain par b(3) ci(3) label replace

reg z_score i.byear NC IUI if subject_grade==2, cl(mor_id)

eststo e1

reg z_score i.byear NC IUI twin_plus i.parity i.b2.maternal_bmi smoker male if subject_grade==2, cl(mor_id)

eststo e2

reg z_score i.byear NC IUI twin_plus i.b4.maternal_age i.parity i.b2.maternal_bmi smoker partner i.level i.ie_type dispo disp_ young old male if subject_grade==2, cl(mor_id)

eststo e3

esttab e* using $data/tables/grade2_IUI.rtf, plain par b(3) ci(3) label replace

esttab e*, keep(IUI) ci(3) b(3)

reg z_score i.byear NC IUI if subject_grade==3, cl(mor_id)

eststo e1

reg z_score i.byear NC IUI twin_plus i.parity i.b2.maternal_bmi smoker male if subject_grade==3, cl(mor_id)

eststo e2

reg z_score i.byear NC IUI twin_plus i.b4.maternal_age i.parity i.b2.maternal_bmi smoker partner i.level i.ie_type dispo disp_ young old male if subject_grade==3, cl(mor_id)

eststo e3

esttab e* using $data/tables/grade3_IUI.rtf, plain par b(3) ci(3) label replace

********************************************************************************

********************************************************************************

** PARITY 1

**

********************************************************************************

********************************************************************************

snapshot erase _all

snapshot save

keep if parity ==1

reg z_score i.byear i.b3.treated if subject_grade==2, cl(mor_id)

eststo e1

margins treated

reg z_score i.byear i.b3.treated twin_plus i.b2.maternal_bmi smoker male if subject_grade==2, cl(mor_id)

eststo e2

margins treated

reg z_score i.byear i.b3.treated twin_plus i.b4.maternal_age i.b2.maternal_bmi smoker partner i.level i.ie_type dispo disp_ young old male if subject_grade==2, cl(mor_id)

eststo e3

margins treated

esttab e* using $data/tables/grade2_both_par1.rtf, plain par b(3) ci(3) label replace

reg z_score i.byear i.b3.treated if subject_grade==3, cl(mor_id)

eststo e1

margins treated

reg z_score i.byear i.b3.treated twin_plus i.b2.maternal_bmi smoker male if subject_grade==3, cl(mor_id)

eststo e2

margins treated

reg z_score i.byear i.b3.treated twin_plus i.b4.maternal_age i.b2.maternal_bmi smoker partner i.level i.ie_type dispo disp_ young old male if subject_grade==3, cl(mor_id)

eststo e3

margins treated

esttab e* using $data/tables/grade3_both_par1.rtf, plain par b(3) ci(3) label replace

reg z_score i.byear NC IUI if subject_grade==2, cl(mor_id)

eststo e1

reg z_score i.byear NC IUI twin_plus i.b2.maternal_bmi smoker male if subject_grade==2, cl(mor_id)

eststo e2

reg z_score i.byear NC IUI twin_plus i.b4.maternal_age i.b2.maternal_bmi smoker partner i.level i.ie_type dispo disp_ young old male if subject_grade==2, cl(mor_id)

eststo e3

esttab e* using $data/tables/grade2_IUI_par1.rtf, plain par b(3) ci(3) label replace

esttab e*, keep(IUI) ci(3) b(3)

reg z_score i.byear NC IUI if subject_grade==3, cl(mor_id)

eststo e1

reg z_score i.byear NC IUI twin_plus i.parity i.b2.maternal_bmi smoker male if subject_grade==3, cl(mor_id)

eststo e2

reg z_score i.byear NC IUI twin_plus i.b4.maternal_age i.parity i.b2.maternal_bmi smoker partner i.level i.ie_type dispo disp_ young old male if subject_grade==3, cl(mor_id)

eststo e3

esttab e* using $data/tables/grade3_IUI_par1.rtf, plain par b(3) ci(3) label replace

*******************************************************************************

*******************************************************************************

** Keep singleton birth

*******************************************************************************

*******************************************************************************

snapshot use 1

keep if twin_ ==0

reg z_score i.byear i.b3.treated if subject_grade==2, cl(mor_id)

eststo e1

margins treated

reg z_score i.byear i.b3.treated i.parity twin_plus i.b2.maternal_bmi smoker male if subject_grade==2, cl(mor_id)

eststo e2

margins treated

reg z_score i.byear i.b3.treated i.parity twin_plus i.b4.maternal_age i.b2.maternal_bmi smoker partner i.level i.ie_type dispo disp_ young old male if subject_grade==2, cl(mor_id)

eststo e3

margins treated

esttab e* using $data/tables/grade2_both_singleton1.rtf, plain par b(3) ci(3) label replace

reg z_score i.byear i.b3.treated if subject_grade==3, cl(mor_id)

eststo e1

margins treated

reg z_score i.byear i.b3.treated i.parity twin_plus i.b2.maternal_bmi smoker male if subject_grade==3, cl(mor_id)

eststo e2

margins treated

reg z_score i.byear i.b3.treated i.parity twin_plus i.b4.maternal_age i.b2.maternal_bmi smoker partner i.level i.ie_type dispo disp_ young old male if subject_grade==3, cl(mor_id)

eststo e3

margins treated

esttab e* using $data/tables/grade3_both_singleton1.rtf, plain par b(3) ci(3) label replace

reg z_score i.byear NC IUI if subject_grade==2, cl(mor_id)

eststo e1

reg z_score i.byear NC IUI i.parity twin_plus i.b2.maternal_bmi smoker male if subject_grade==2, cl(mor_id)

eststo e2

reg z_score i.byear NC IUI i.parity twin_plus i.b4.maternal_age i.b2.maternal_bmi smoker partner i.level i.ie_type dispo disp_ young old male if subject_grade==2, cl(mor_id)

eststo e3

esttab e* using $data/tables/grade2_IUI_singleton1.rtf, plain par b(3) ci(3) label replace

esttab e*, keep(IUI) ci(3) b(3)

reg z_score i.byear NC IUI if subject_grade==3, cl(mor_id)

eststo e1

reg z_score i.byear NC IUI twin_plus i.parity i.b2.maternal_bmi smoker male if subject_grade==3, cl(mor_id)

eststo e2

reg z_score i.byear NC IUI i.parity twin_plus i.b4.maternal_age i.parity i.b2.maternal_bmi smoker partner i.level i.ie_type dispo disp_ young old male if subject_grade==3, cl(mor_id)

eststo e3

esttab e* using $data/tables/grade3_IUI_singleton1.rtf, plain par b(3) ci(3) label replace

*****************************************************

**

** ** ONLY 2007+

**

*********************************************************

snapshot use 1

reg z_score i.byear i.b3.treated if subject_grade==2 & byear > 2006, cl(mor_id)

eststo e1

margins treated

reg z_score i.byear i.b3.treated twin_plus i.parity i.b2.maternal_bmi smoker male if subject_grade==2 & byear > 2006, cl(mor_id)

eststo e2

margins treated

reg z_score i.byear i.b3.treated twin_plus i.b4.maternal_age i.parity i.b2.maternal_bmi smoker partner i.level i.ie_type dispo disp_ young old male if subject_grade==2 & byear > 2006, cl(mor_id)

eststo e3

margins treated

esttab e* using $data/tables/grade2_both_2007.rtf, plain par b(3) ci(3) label replace

reg z_score i.byear art iui twin_plus i.b4.maternal_age i.parity i.b2.maternal_bmi smoker partner i.level i.ie_type dispo disp_ young old male if subject_grade==2 & byear > 2006, cl(mor_id)

reg z_score i.byear i.b3.treated if subject_grade==3 & byear > 2006, cl(mor_id)

eststo e1

margins treated

reg z_score i.byear i.b3.treated twin_plus i.parity i.b2.maternal_bmi smoker male if subject_grade==3 & byear > 2006, cl(mor_id)

eststo e2

margins treated

reg z_score i.byear i.b3.treated twin_plus i.b4.maternal_age i.parity i.b2.maternal_bmi smoker partner i.level i.ie_type dispo disp_ young old male if subject_grade==3 & byear > 2006, cl(mor_id)

eststo e3

margins treated

esttab e* using $data/tables/grade3_both_2007.rtf, plain par b(3) ci(3) label replace

reg z_score i.byear NC IUI if subject_grade==2 & byear > 2006, cl(mor_id)

eststo e1

reg z_score i.byear NC IUI twin_plus i.parity i.b2.maternal_bmi smoker male if subject_grade==2 & byear > 2006, cl(mor_id)

eststo e2

reg z_score i.byear NC IUI twin_plus i.b4.maternal_age i.parity i.b2.maternal_bmi smoker partner i.level i.ie_type dispo disp_ young old male if subject_grade==2 & byear > 2006, cl(mor_id)

eststo e3

esttab e* using $data/tables/grade2_IUI_2007.rtf, plain par b(3) ci(3) label replace

esttab e*, keep(IUI) ci(3) b(3)

reg z_score i.byear NC IUI if subject_grade==3 & byear > 2006, cl(mor_id)

eststo e1

reg z_score i.byear NC IUI twin_plus i.parity i.b2.maternal_bmi smoker male if subject_grade==3 & byear > 2006, cl(mor_id)

eststo e2

reg z_score i.byear NC IUI twin_plus i.b4.maternal_age i.parity i.b2.maternal_bmi smoker partner i.level i.ie_type dispo disp_ young old male if subject_grade==3 & byear > 2006, cl(mor_id)

eststo e3

esttab e* using $data/tables/grade3_IUI_2007.rtf, plain par b(3) ci(3) label replace

********************************************************************************

** By education

**

********************************************************************************

snapshot use 1

reg z_score i.byear i.b3.treated twin_plus i.b4.maternal_age i.parity i.b2.maternal_bmi smoker partner i.ie_type i.level dispo disp_ young old male if subject_grade==2 & level <= 2, cl(mor_id)

eststo e1

margins treated

reg z_score i.byear NC IUI twin_plus i.b4.maternal_age i.parity i.b2.maternal_bmi smoker partner i.ie_type i.level dispo disp_ young old male if subject_grade==2 & level <= 2, cl(mor_id)

reg z_score i.byear i.b3.treated twin_plus i.b4.maternal_age i.parity i.b2.maternal_bmi smoker partner i.ie_type dispo disp_ young old male if subject_grade==2 & level > 2, cl(mor_id)

eststo e2

margins treated

reg z_score i.byear NC IUI twin_plus i.b4.maternal_age i.parity i.b2.maternal_bmi smoker partner i.ie_type dispo disp_ young old male if subject_grade==2 & level > 2, cl(mor_id)

reg z_score i.byear i.b3.treated twin_plus i.b4.maternal_age i.parity i.b2.maternal_bmi smoker partner i.ie_type i.level dispo disp_ young old male if subject_grade==3 & level <= 2, cl(mor_id)

eststo e3

margins treated

reg z_score i.byear NC IUI twin_plus i.b4.maternal_age i.parity i.b2.maternal_bmi smoker partner i.ie_type i.level dispo disp_ young old male if subject_grade==3 & level <= 2, cl(mor_id)

reg z_score i.byear i.b3.treated twin_plus i.b4.maternal_age i.parity i.b2.maternal_bmi smoker partner i.ie_type dispo disp_ young old male if subject_grade==3 & level > 2, cl(mor_id)

eststo e4

margins treated

reg z_score i.byear NC IUI twin_plus i.b4.maternal_age i.parity i.b2.maternal_bmi smoker partner i.ie_type dispo disp_ young old male if subject_grade==3 & level > 2, cl(mor_id)

esttab e* using $data/tables/grade2_3edu.rtf, plain par b(3) ci(3) label replace mtitle("Low edu, grade 2" "High edu, grade 2" "Low edu, grade 3" "High edu, grade3")
